# HOXA5-mediated spatial remodeling of tumor-immune interfaces across cancers promotes AML pathogenesis

**DOI:** 10.3389/fimmu.2025.1677713

**Published:** 2025-10-23

**Authors:** Changling Zhang, Ping Wen, Yan Zeng, Tao Chen, Qulian Guo, Chunyan Liu, Fangfang Zhong

**Affiliations:** ^1^ Pediatric Department of Urology and Immunology, Children’s Medical Center, The Affiliated Hospital of Southwest Medical University, Luzhou, Sichuan, China; ^2^ Sichuan Clinical Research Center for Birth Defects, Luzhou, Sichuan, China

**Keywords:** hoxa5, cell proliferation, cell cycle, AML, biomarkers

## Abstract

**Background:**

HOXA5 (homeobox A5) exhibits context-dependent roles in cancer, but its pan-cancer spatial immune regulatory functions and therapeutic potential remain poorly understood.

**Methods:**

We integrated multi-omics data from 33 cancer types (TCGA, n=11,096; GTEx, n=7,469; TISCH2; spatial transcriptomics) to characterize HOXA5 expression, genomic alterations, and immune interactions. Functional validation was performed in AML cell lines (U937, KG-1; n=3 biological replicates per experiment).

**Results:**

HOXA5 was significantly dysregulated across cancers, with elevated expression in AML and GBM, and reduced expression in BRCA and LUAD. In AML, high HOXA5 expression predicted poor overall survival (HR = 2.80, 95% CI: 1.60–4.89, p < 0.001) and was associated with FLT3/NPM1 mutations. Spatial transcriptomics revealed HOXA5+ malignant cells enhance fibroblast/endothelial crosstalk via IGFBP3-TMEM219. HOXA5 knockdown suppressed proliferation (p < 0.01) and induced G0/G1 arrest. Mechanistically, HOXA5 maintained AML through cholesterol biosynthesis and ECM remodeling. Mercaptopurine was identified as a potential therapeutic agent, and molecular docking predicted a potential stable interaction with HOXA5.

**Conclusions:**

HOXA5 plays a dual role in solid versus hematologic malignancies and serves as a key spatial immune regulator. It is a robust prognostic biomarker and therapeutic target in AML, with mercaptopurine representing a promising repurposing candidate.

## 1 Introduction

Cancer remains a leading cause of death worldwide, characterized by extensive molecular heterogeneity and complex tumor microenvironment interactions (1, 2). Despite advances in targeted therapies and immune checkpoint inhibitors, treatment resistance driven by genomic instability, epigenetic dysregulation, and immunosuppressive factors continues to limit clinical efficacy (3–5). Consequently, identifying reliable pan-cancer biomarkers for patient stratification, treatment response prediction, and novel biological mechanism discovery is crucial for precision oncology.

The HOXA5 gene encodes a homeobox transcription factor essential for embryonic development and tissue patterning (6). Previous studies indicate that HOXA5 acts as a tumor suppressor by inhibiting proliferation and inducing apoptosis. Specifically, HOXA5 directly activates p53 transcription by binding to its promoter ATTA motif, upregulating p21/Caspase-3 to induce G1/S cell cycle arrest and apoptosis in cervical cancer (7–9). In p53-mutant breast cancer cells, HOXA5 induces apoptosis via Caspase-2/8 activation (10). Paradoxically, it promotes tumor progression in glioblastoma (11). This functional duality, coupled with regulation by DNA methylation and copy number variation, positions HOXA5 as a compelling biomarker candidate (12). However, systematic analyses of its pan-cancer expression patterns, spatial immune regulation, and therapeutic significance—particularly within tumor-immune co-evolutionary contexts—remain lacking.

To address these gaps, we conducted a comprehensive multi-omics analysis of HOXA5 across 33 cancer types. We integrated single-cell transcriptomics, spatial transcriptomics, epigenomics, and pharmacogenomics data from The Cancer Genome Atlas (TCGA), Genotype-Tissue Expression (GTEx), Tumor Immune Single-cell Hub 2 (TISCH2), and other public databases. Functional validation in acute myeloid leukemia (AML) cells elucidated its mechanistic role in proliferation and cell cycle regulation. Our study aims to: (1) reveal pan-cancer dysregulation of HOXA5 and its genomic determinants; (2) characterize its spatially resolved crosstalk with the tumor immune ecosystem; (3) establish its clinical utility as an independent prognostic biomarker in AML; and (4) identify targeted therapies against HOXA5-driven tumorigenesis. This work provides a foundation for precise HOXA5-targeted interventions.

## 2 Materials and methods

### 2.1 Datasets acquisition

Raw RNA-seq and clinical data were obtained from the PanCancer Atlas publication page (https://gdc.cancer.gov/about-data/publications/pancanatlas). To improve the reliability of differential expression, we paired the TPM expression levels of GTEx normal samples with the TPM expression levels of TCGA tumors (from the tcga_RSEM_gene_tpm and gtex_RSEM_gene_tpm datasets in the USCS Xena database). To minimize anatomical confounding, only TCGA primary tumor tissues were retained for pairing with GTEx data, and Z-scores were used for data standardization. Pan-cancer transcript, methylation, and copy number variation data were sourced from the UCSC Xena platform (https://xenabrowser.net/). Pan-cancer expression quantitative trait loci-Genome Wide Association Study (eQTL-GWAS) colocalization data were acquired from Open GWAS (https://gwas.mrcieu.ac.uk/). scRNA-seq data were obtained from TISCH2 (http://tisch.comp-genomics.org/) (13). Cancer-Immunity Cycle data were sourced from the Tracking Tumor Immunophenotype (TIP; http://biocc.hrbmu.edu.cn/TIP/) database (14). Pan-cancer immune cell infiltration data were downloaded from the Tumor Immunity Assessment Resource 2.0 (TIMER2.0; http://timer.cistrome.org/) (15). 24 immune cell subtype markers were derived from published studies (16). Reverse phase protein array data were obtained from The Cancer Proteome Atlas (TCPA; http://www.tcpaportal.org). Functional states of 14 tumor cell types were acquired from CancerSEA (http://biocc.hrbmu.edu.cn/CancerSEA/home.jsp) (17). Spatial transcriptome data for BRCA (GSE210616-GSM6433596) (18), GIST (GSE203612-GSM6177607) (19), and OV (GSE211956-GSM6506112) (20) were obtained from GEO (https://www.ncbi.nlm.nih.gov/geo/). PRAD spatial transcriptome data were sourced from 10x Genomics (https://www.10xgenomics.com/), while CRC and LIHC data were from previous studies (21, 22). The abbreviations for all cancers can be found in **Supplementary Table S1**.

### 2.2 Single-cell and spatial transcriptomic data analysis

The R package pheatmap visualized pan-cancer single-cell expression profiles. Uniform manifold approximation and projection (UMAP) was used for dimensionality reduction. Cell-cell communication was assessed using the CellChat package (23). The Cottrazm package within SpatialTME deconvolved tumor microenvironment cell composition (24, 25). For each microregion, the predominant cell type was identified, and the Seurat package’s SpatialDimPlot visualized top cell compositions and HOXA5 expression. Spearman correlation assessed associations between cell content across all points and between cell content and HOXA5 expression, visualized using linkET. Microregions with >0% malignant cells were classified as malignant (Mal); those with 0% malignant cells were non-malignant (nMal) (**Supplementary Figure S2A**). Wilcoxon tests compared expression differences between groups, with bar graphs displaying mean expression levels.

### 2.3 Characterization of HOXA5 expression and mutational spectrum across Malignancies

HOXA5 expression in normal human tissues was evaluated using GTEx data. Expression in immune cells and tumor cell lines was similarly assessed. Differential HOXA5 expression between tumor and normal tissues was evaluated using TCGA pan-cancer expression data and validated in additional datasets. Gene expression values (TPM) were normalized to Z-scores within each cancer type to enable cross-sample comparison. Outliers with extreme expression (|Z| > 3 for most genes) were excluded. Cancer types retaining at least three normal samples after filtration were included in subsequent differential expression analysis. Logistic regression assessed associations between HOXA5 expression and tumor status (tumor vs. normal).

HOXA5 mutation, amplification, and deletion frequencies were analyzed using cBioPortal. Copy number variation (CNV) was evaluated using pan-cancer CNV data, with Spearman correlation assessing HOXA5 CNV-mRNA expression relationships. Associations between HOXA5 genomic status and immune response were evaluated following Thorsson et al. (26). Differences in methylation levels of HOXA5 probes between normal and tumor tissues were assessed using 450K methylation data, with Spearman correlation evaluating methylation-mRNA expression relationships. The methylation module of Tumor Immune Dysfunction and Exclusion (TIDE; http://tide.dfci.harvard.edu/) evaluated HOXA5-prognosis correlations (27). Bayesian colocalization analysis was performed to assess whether two traits share the same causal genetic variant using the “coloc” R package (v5.2.3) with default prior probabilities (p1 = 1×10^-4^, p2 = 1×10^-4^, p12 = 1×10^-5^). A genomic window of ±100 kb around the HOXA5 locus was defined for the analysis. Linkage disequilibrium (LD) reference was derived from the 1000 Genomes Project European population data. The ieugwasr_to_coloc function was used to extract colocalization data, and the coloc.abf function was applied to test for shared causal variants between the eQTL signals of HOXA5 and cancer risk GWAS traits. Colocalization evidence was considered significant if the posterior probability for H4 (PP.H4.abf) exceeded 80%. Visualization of the colocalized region was performed using the stack_assoc_plot function from the gassocplot2 R package.

### 2.4 Functional enrichment analysis

HOXA5-interacting genes were identified using GeneMANIA (http://genemania.org/) (28). Protein interaction networks were explored via STRING (https://cn.string-db.org/). Samples with top/bottom 30% HOXA5 expression were defined as high/low expression groups. Differential analysis used the limma package. Gene set enrichment analysis (GSEA) based on hallmark and KEGG metabolic gene sets was performed using clusterProfiler (29). The GSVA package evaluated Pearson correlations between HOXA5 and functional states in 14 tumor cell types (30). Spearman correlation between HOXA5 and TCPA functional protein content was calculated using cor.test. Associations between HOXA5 and 10 cancer-related pathway scores were evaluated as described (31).

### 2.5 Immunological correlation analysis

Stromal and immune scores were calculated using the ESTIMATE package (32). Immune infiltration in AML was quantified using the CIBERSORT algorithm (https://cibersortx.stanford.edu/) with 22 immune cell markers. Single-sample GSEA (ssGSEA) evaluated immune infiltration. Spearman correlation assessed gene-TIP score relationships and TIP score autocorrelations, visualized with linkET. MeTIL signature scores were derived via principal component analysis (PCA) of methylation values (33), standardized as Z-scores [(x - μ)/σ]. IFNγ response scores and T-cell-inflamed scores in AML were computed using the easier R package (34), which leverages validated gene signatures derived from transcriptomic profiles of patients treated with immune checkpoint inhibitors. Differences between HOXA5 expression groups were assessed using the Wilcoxon rank-sum test.

### 2.6 Association of HOXA5 with AML clinical features

Logistic regression (stats package) evaluated associations between HOXA5 and AML clinical variables. Kaplan-Meier survival analysis used the survival package. Optimal expression cutoffs were determined using survminer (minimum group proportion ≥0.3). Survival differences were assessed with log-rank tests. Patients were quartiled (Q1: highest 25%, Q4: lowest 25%) by HOXA5 expression; chi-square tests evaluated group composition differences. Restricted cubic splines (RCS) explored nonlinear survival risk relationships. The timeROC package evaluated HOXA5’s predictive value for 1-, 3-, and 5-year AML survival. Univariate Cox analysis described hazard ratios (HR) and 95% confidence intervals (CI). The rms package constructed nomograms, calibration plots, and decision curve analysis (DCA). HOXA5’s prognostic value was further validated using the Kaplan-Meier plotter database (https://kmplot.com/analysis/index.php?p=home).

### 2.7 Drug sensitivity analysis

Correlations between HOXA5 and drug sensitivity were evaluated using the CellMiner database (35). Spearman correlation assessed relationships between HOXA5 expression and dose-response (AUC) values in the PRISM database (36). Connectivity Map (cMAP) analysis identified compounds potentially counteracting HOXA5-mediated oncogenesis using the XSum method (37, 38), with lower scores indicating potential inhibitors. The SWISS-MODEL server (https://swissmodel.expasy.org/) generated HOXA5 protein structures (39). Small molecule structures were downloaded from PubChem (https://pubchem.ncbi.nlm.nih.gov/). Molecular docking used CB-Dock2 (http://cadd.labshare.cn/cb-dock2/php/index.php), visualized with PyMOL (40).

### 2.8 Cell culture

U937 and KG-1 cells (Chinese Academy of Sciences Cell Bank, Shanghai) were cultured at 37 °C with 5% CO_2_. U937 cells were maintained in RPMI-1640 (Gibco) supplemented with 10% FBS (Vazyme), 100 μg/mL streptomycin, and 100 U/mL penicillin. KG-1 cells were grown in IMDM (Gibco) with 20% FBS (Vazyme), 100 μg/mL streptomycin, and 100 U/mL penicillin.

### 2.9 Quantitative real-time PCR

Total RNA was extracted using TRIzol (Invitrogen). cDNA was synthesized from 1 μg RNA (Hiscript III cDNA kit, Vazyme). GAPDH served as a control. Reactions contained: 1 μL cDNA, 0.6 μL forward/reverse primers (10 μM), 7.5 μL ChamQ SYBR qPCR Master Mix (Vazyme), and 6.3 μL ddH_2_O. Cycling conditions: 95 °C for 10 min; 40 cycles of 95 °C for 15 s, 62 °C for 1 min, 72 °C for 15 s; final extension: 60 °C for 1 min, 95 °C for 15 s. Primers: GAPDH-F: GGAGCGAGATCCCTCCAAAAT; GAPDH-R: GGCTGTTGTCATACTTCTCATGG; HOXA5-F: ACCCACATCAGCAGCAGAGA; HOXA5-R: GGCCGCCTATGTTGTCAT.

### 2.10 Western blotting

Cells were harvested post-siRNA treatment, washed with PBS, and lysed in RIPA buffer with protease inhibitors (Solarbio). Proteins were separated by SDS-PAGE, transferred to membranes, and probed with anti-HOXA5 (Abcam) and anti-GAPDH (Proteintech) antibodies. Goat Anti-Rabbit IgG-HRP (Proteintech) was the secondary antibody. Signals were detected using ECL (4A Biotech).

### 2.11 Cell viability assay

Viability was assessed using CCK-8 (APExBIO). siRNA-transfected U937 and KG-1 cells (60% confluency) were seeded in 96-well plates (5,000 cells/well, 5 replicates/group). Absorbance (450 nm) was measured at 0, 24, 48, and 72 h.

### 2.12 Cell cycle analysis

Cell cycle distribution was analyzed using a Cell Cycle Detection Kit (KeyGen Biotech). Cells were fixed in 70% cold ethanol (4 °C overnight), washed with PBS, stained with PI/RNase A for 30 min, and analyzed by flow cytometry (Beckman CytExpert).

### 2.13 RNA sequencing

RNA sequencing (RNA-seq) was performed by MetWare Biotechnology (Wuhan, China). Differential expression analysis between comparison groups was conducted using the DESeq2 package applied to raw read counts. The resulting P-values were adjusted for multiple testing using the Benjamini-Hochberg method to control the False Discovery Rate (FDR). Differentially expressed genes (DEGs) were defined as those with |log2Fold Change| ≥ 1 and an adjusted P-value (FDR) < 0.05. Functional enrichment used Metascape (https://metascape.org/).

### 2.14 Statistical analysis

R v4.2.1 and online databases were used. Group differences were assessed by Wilcoxon rank-sum (two groups) or Kruskal-Wallis (≥3 groups) tests. Survival analyses employed Kaplan-Meier curves with log-rank tests or Cox regression. Relationships between variables were evaluated using Pearson/Spearman correlation. Cellular experiments were performed in triplicate; data are mean ± SD. GraphPad Prism v9.0.0 analyzed cellular data. Significance: *p < 0.05, **p < 0.01, ***p < 0.001, ns (not significant).

## 3 Results

### 3.1 HOXA5 is significantly dysregulated across cancers

**Figure 1:**
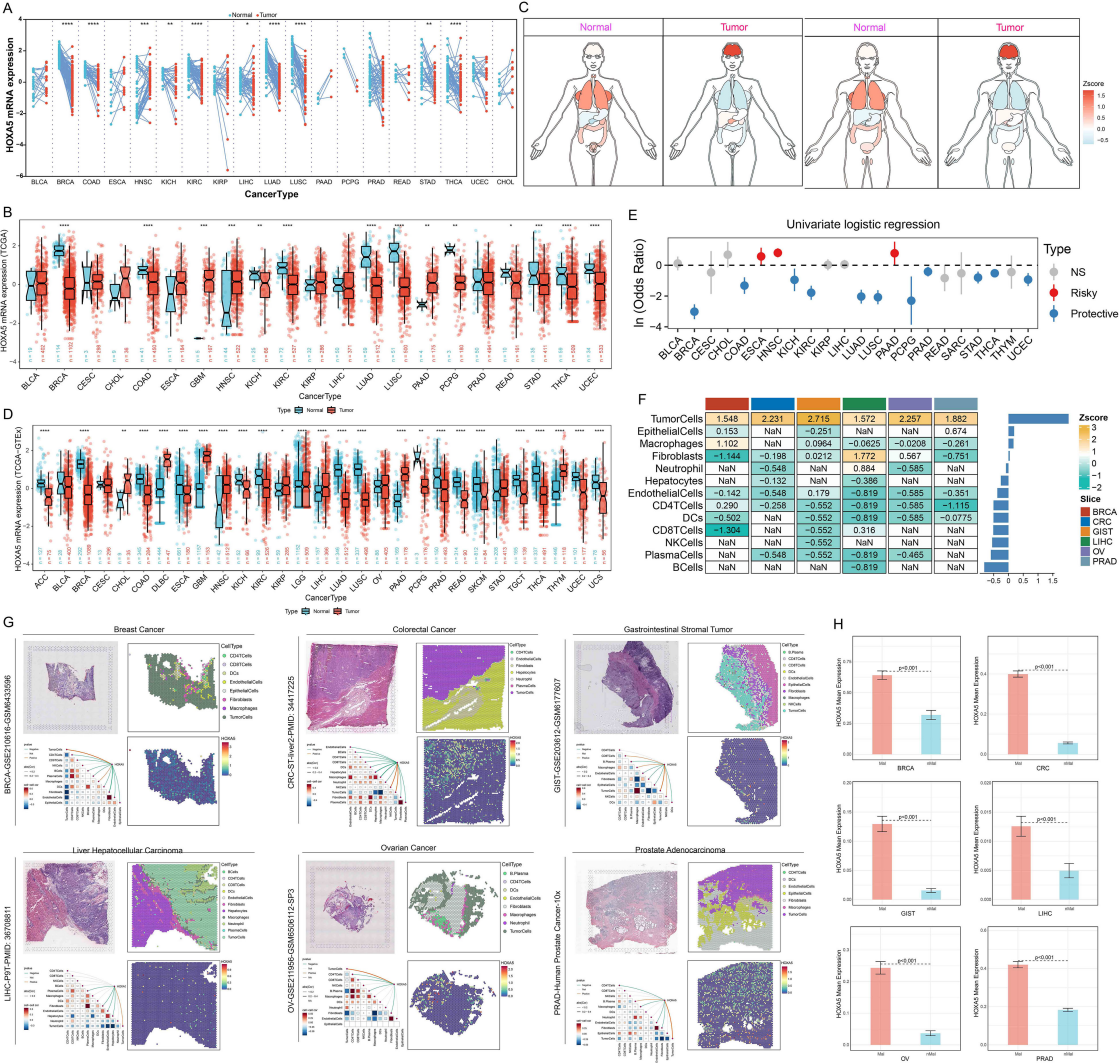
HOXA5 expression is dysregulated across cancers and enriched in malignant niches. **(A)** HOXA5 expression in TCGA tumor vs. paired normal tissues; **(B)** HOXA5 expression in TCGA tumor vs. unpaired normal tissues; **(C)** Organ-specific HOXA5 expression differences (tumor vs. normal); **(D)** HOXA5 expression in tumor (TCGA) and normal (GTEx) tissues; **(E)** Logistic regression of the association between HOXA5 expression and tumor status; **(F)** Heatmap of HOXA5 expression across cell-type-specific microdomains in spatial transcriptomics data; **(G)** Spatial co-localization of HOXA5 expression with malignant cell clusters; **(H)** HOXA5 expression in malignant (Mal) vs. non-malignant (nMal) microregions. *p < 0.05, **p < 0.01, ***p < 0.001; ns: not significant.

Analysis of GTEx data revealed high HOXA5 expression in normal adrenal gland, fallopian tube, and kidney tissues, and in immune cells (e.g., basophils, NK cells; **Supplementary Figure S1B**). Tumor cell lines (e.g., uterine, kidney, colorectal cancer) also showed elevated HOXA5 expression (**Supplementary Figure S1C**). Pan-cancer transcriptome analysis indicated significantly high HOXA5 expression in GBM and LAML, but low expression in BRCA and LUAD (**Supplementary Figure S1D**). Examination of TCGA tumor/normal pairs confirmed significant HOXA5 dysregulation in BRCA, HNSC, and LUAD (**Figures 1A, B**). Integration with GTEx normal samples revealed dysregulation in >75% of tumor types: significant downregulation in BRCA, LUAD, LUSC, and READ; significant upregulation in DLBC, GBM, and PAAD (**Figures 1C, D**). Validation using the GENT2 database supported these findings (**Supplementary Figure S1E**) (41). Regression analysis (TCGA) confirmed significant associations between HOXA5 and tumor status (**Figure 1E**). HOXA5 positively correlated with ESCA, HNSC, and PAAD tumor status (high expression increases cancer risk), and negatively correlated with BRCA, COAD, and LUAD status (low expression increases cancer risk). Spatial transcriptomics showed significantly higher HOXA5 expression in malignant microregions (**Figure 1F**). HOXA5 exhibited spatial co-localization with malignant cells and strong positive correlation with tumor cell content, concentrated within malignant subpopulations (**Figure 1G**). Malignant (Mal; >0% malignant cells) and non-malignant (nMal; 0% malignant cells) microregions were defined (**Supplementary Figure S2A**). HOXA5 expression was significantly higher in Mal versus nMal regions (**Figure 1H**). Single-cell analysis confirmed high HOXA5 expression in malignant cells, fibroblasts, and endothelial cells across cancers (**Supplementary Figure S2B**), suggesting its role in tumor progression.

### 3.2 Pan-cancer genomic alterations of HOXA5

**Figure 2:**
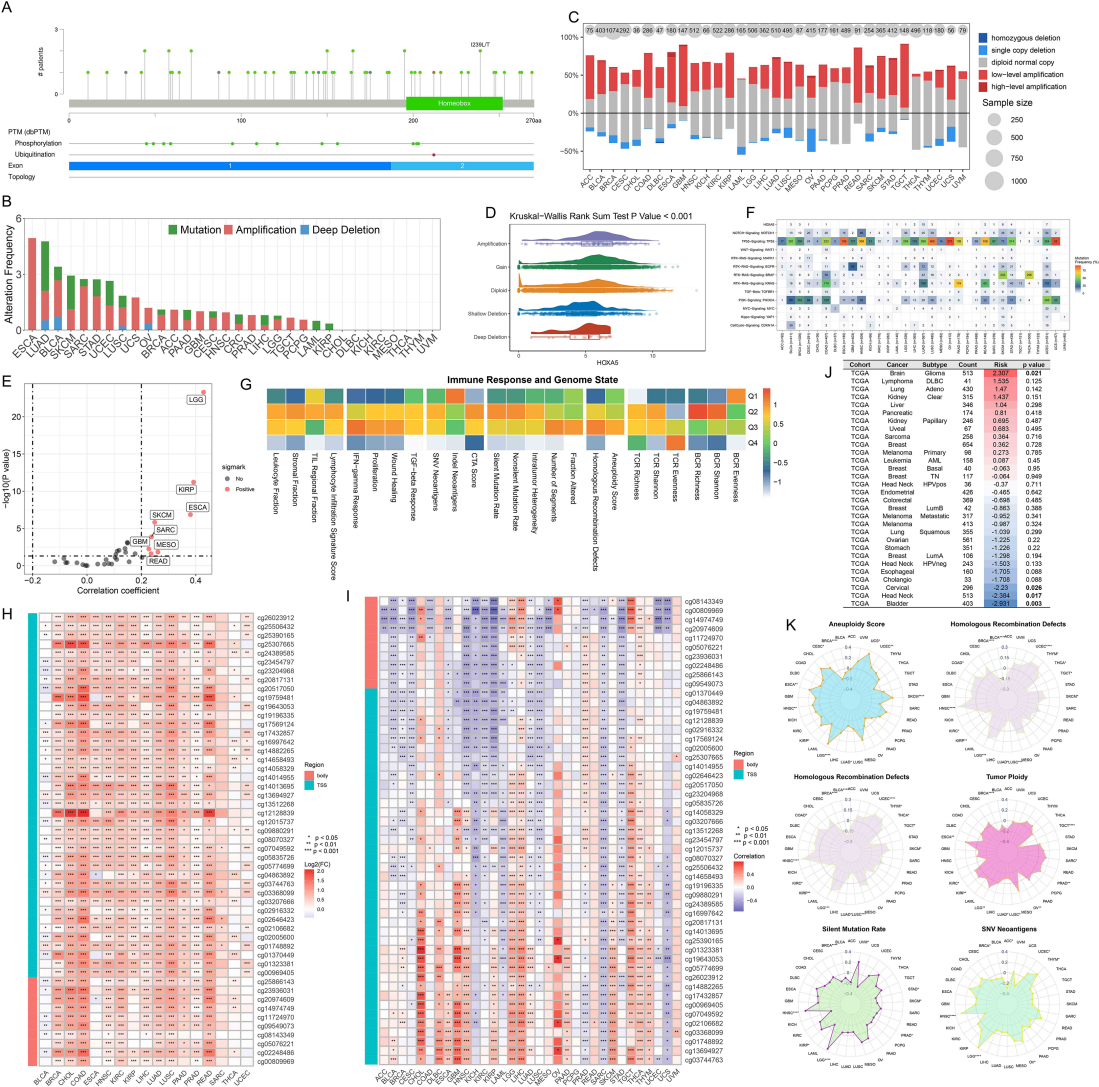
Genomic and epigenetic landscape of HOXA5 across cancers. **(A)** Locations of HOXA5 mutations in pan-cancer analysis; **(B)** Frequency of HOXA5 genetic alterations; **(C)** Copy number variation (CNV) patterns of HOXA5; **(D)** HOXA5 expression stratified by CNV status; **(E)** Correlation between HOXA5 CNV and mRNA expression; **(F)** Co-mutation analysis of HOXA5 and key oncogenic pathways; **(G)** Heatmap of immune response and genomic status scores by HOXA5 expression level; **(H)** Differential methylation of HOXA5 in tumor vs. normal tissues; **(I)** Correlation between HOXA5 methylation and mRNA expression; **(J)** Prognostic value of HOXA5 methylation; **(K)** Radar plot of correlations between HOXA5 expression and genomic status scores. *p < 0.05, **p < 0.01, ***p < 0.001, ****p < 0.0001.

**Figure 3:**
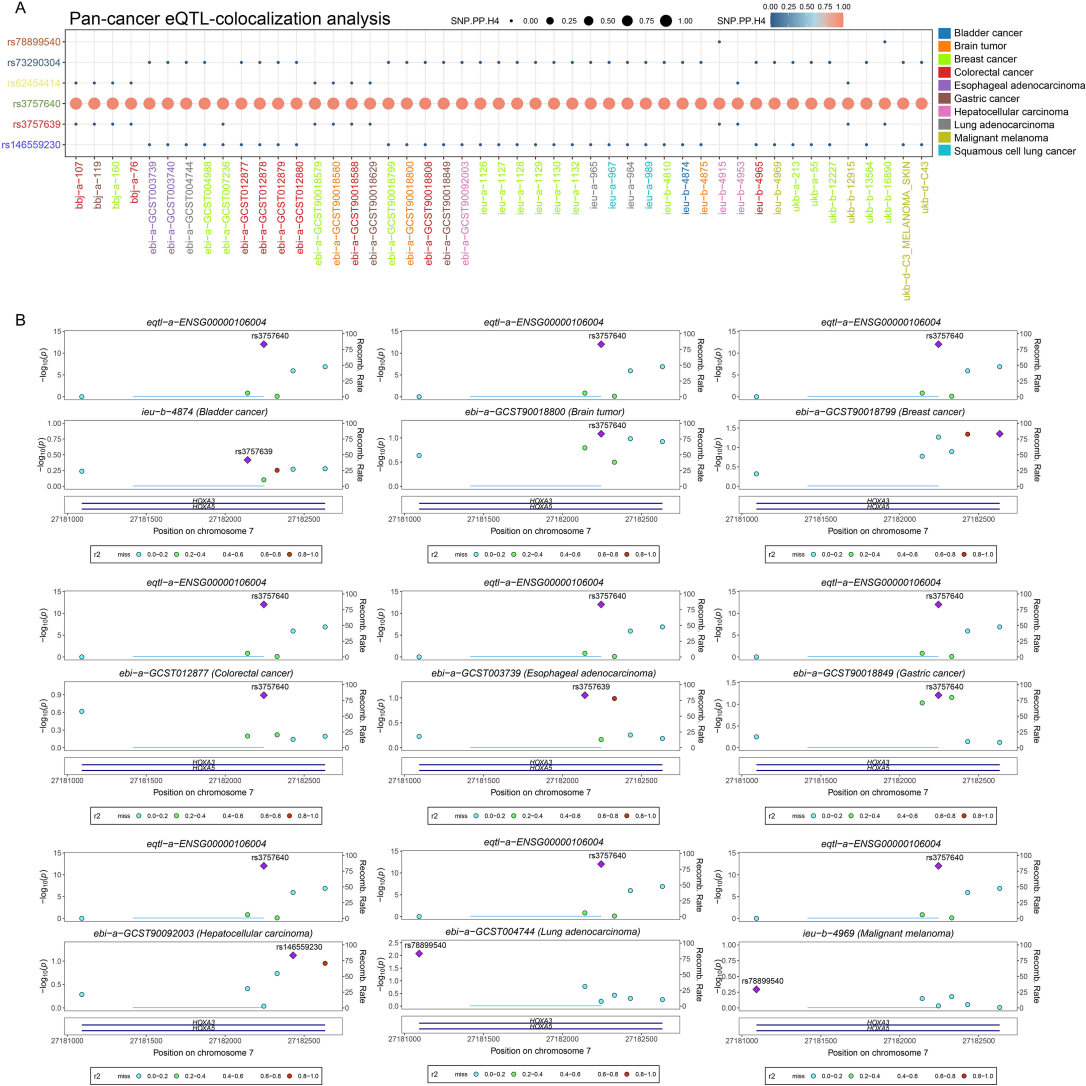
Colocalization of HOXA5 eQTL with cancer risk loci. **(A)** Bayesian colocal analysis suggests variant rs3757640 shares causal variants with cancer risk, with very strong colocalization evidence (PP.H4.abf = 1); **(B)** Colocalization results visualized using the gassocplot package.

cBioPortal analysis revealed HOXA5 mutation sites across cancers (**Figure 2A**, **Supplementary Figure S3A**). Amplification was frequent, particularly in ESCA (**Figure 2B**). Copy number variation analysis indicated widespread low-level amplification, with significant deletions mainly in OV and UCS (**Figure 2C**). HOXA5 expression increased progressively from deletion to amplification (**Figure 2D**). Positive correlations between HOXA5 CNV and mRNA expression were observed in LGG, KIRP, and ESCA (**Figure 2E**). HOXA5 mutation distribution and SNV classifications are shown (**Supplementary Figure S3B**). Comparing HOXA5 to oncogenic pathways revealed TP53 as the most frequently mutated (**Figure 2F**). HOXA5-overexpressing tumors exhibited weaker immune responses (**Figure 2G**). Analysis of methylation patterns showed significant HOXA5 hypermethylation in multiple malignancies (**Figure 2H**). Methylation-expression relationships displayed tumor-type specificity: negative in KICH, LAML, SARC, UCS; positive in CHOL, GBM, TGCT (**Figure 2I**). HOXA5 methylation correlated with prognosis in BLCA, glioma, CESC, and HNSC (**Figure 2J**, **Supplementary Figures S3C-F**). Genomic status scores showed varying correlations with HOXA5 expression (**Figure 2K**). eQTL-GWAS colocalization indicated that rs3757640 (HOXA5) shares genetic variation with cancer risk, providing very strong evidence for colocalization under the specified model (PP.H4.abf = 1; **Figures 3A, B**) (**Supplementary Table S2**).

### 3.3 Functional enrichment of HOXA5 in pan-cancer

**Figure 4:**
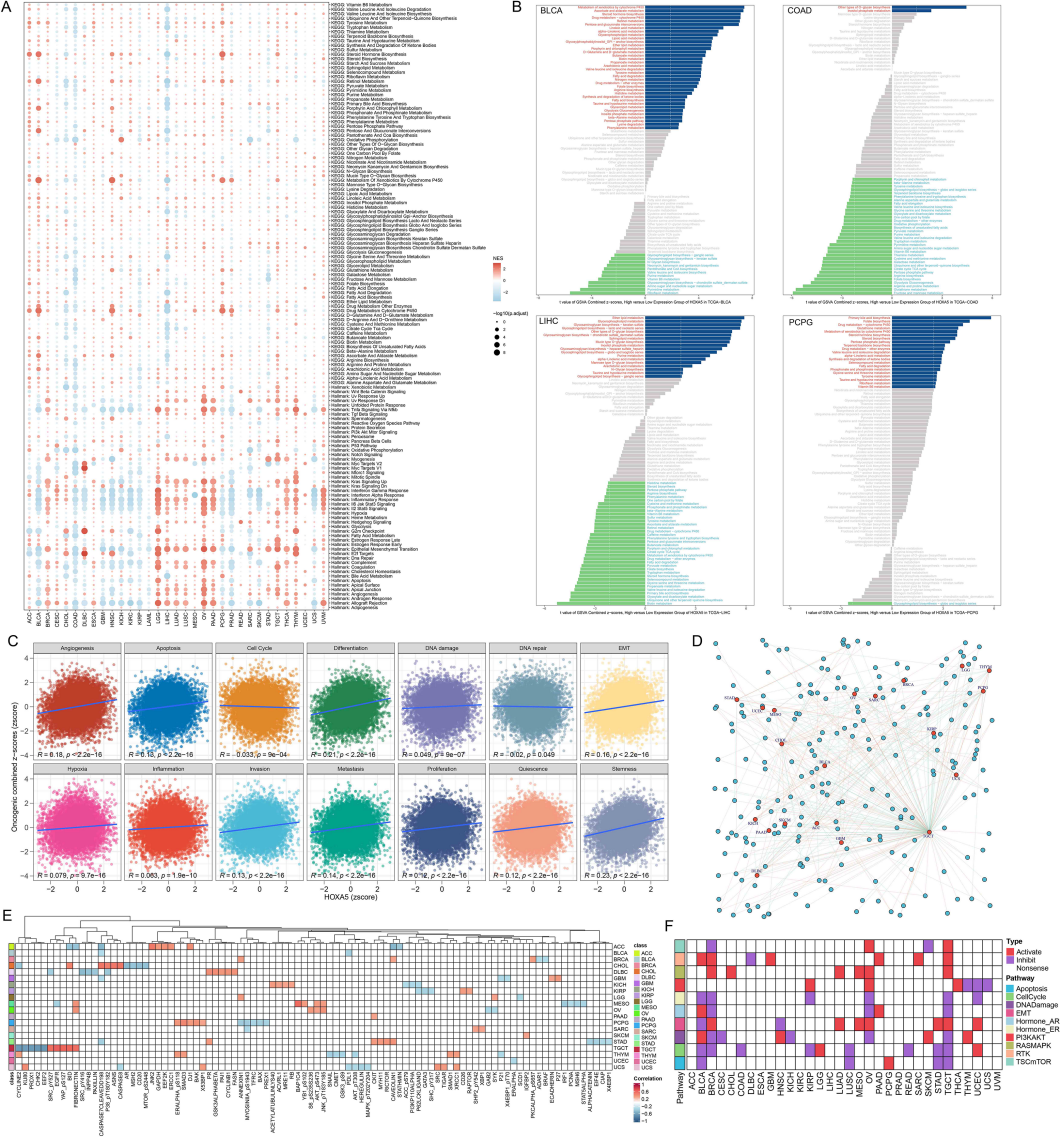
Functional enrichment of HOXA5 in pan-cancer. **(A)** Gene set enrichment analysis (GSEA) of Hallmark and KEGG pathways; **(B)** GSVA scores of metabolic pathways in HOXA5-high vs. -low groups; **(C)** Correlation between HOXA5 expression and malignant functional states; **(D)** Protein interaction network of HOXA5-associated proteins (|correlation| > 0.3, p < 0.05); **(E)** Heatmap of top HOXA5-correlated proteins from the TCPA database; **(F)** Pathway activity differences between HOXA5-high and -low expression groups.

**Figure 5:**
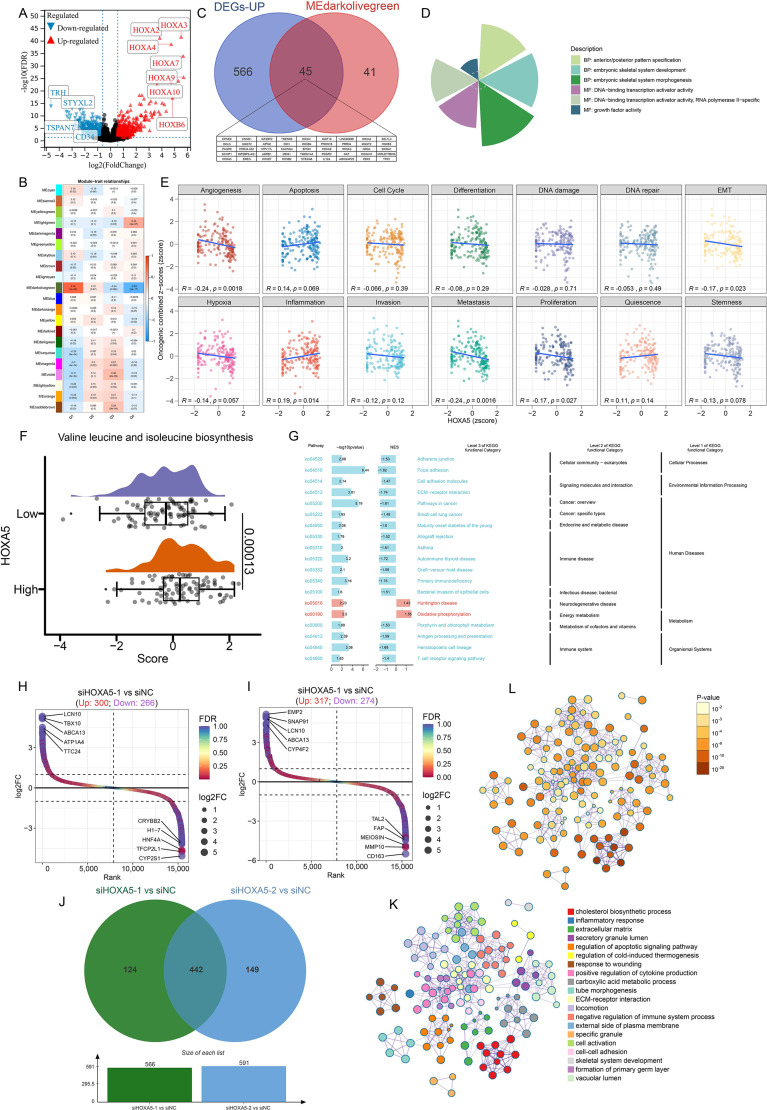
Transcriptomic profiling reveals mechanisms of HOXA5 in AML. **(A)** Volcano plot of differentially expressed genes (DEGs) between HOXA5-high and -low AML patients; **(B)** Module-trait relationships from WGCNA identifying the MEdarkolivegreen module as most correlated with HOXA5; **(C)** Venn diagram identifying 45 core genes overlapping between DEGs and the key WGCNA module; **(D)** Gene Ontology (GO) enrichment analysis of HOXA5-associated genes; **(E)** Correlation between HOXA5 expression and functional states in AML (CancerSEA); **(F)** GSVA of valine, leucine, and isoleucine biosynthesis pathway activity; **(G)** GSEA enrichment plots for selected pathways in HOXA5-low AML; **(H, I)** Volcano plots of DEGs in KG-1 cells after transfection with siHOXA5-1 **(H)** or siHOXA5-2 **(I)**; **(J)** Venn diagram of DEGs from two siRNA treatments, identifying 442 core genes; **(K, L)** Functional enrichment analysis of the 442 core DEGs using Metascape.

HOXA5 exhibited diverse functional states across tumors (**Figure 4A**). Significant negative correlations with immune-related pathways occurred in BLCA, COAD, GBM, HNSC, PRAD; positive correlations occurred in LGG, OV, UVM. HOXA5 also correlated with EMT and Tnfa Signaling Via Nfkb pathways in multiple cancers. Metabolic analysis revealed activation of Steroid Hormone Biosynthesis and Xenobiotic Metabolism By Cytochrome P450 in HOXA5-high BLCA and PCPG, but inhibition in HOXA5-high COAD and LIHC (**Figure 4B**). GSVA indicated positive correlations between HOXA5 and Angiogenesis, Differentiation, EMT, and Stemness (**Figure 4C**). TCPA analysis identified numerous HOXA5-associated proteins (**Figure 4D**), with the top positively/negatively correlated shown (**Figure 4E**). HOXA5 was significantly associated with oncogenic pathways (**Figure 4F**), highlighting its role in tumor development. It is noteworthy that these positive associations with angiogenesis, EMT, and stemness were derived from our pan-cancer analysis, which is predominantly driven by solid tumors. This stands in contrast to the role of HOXA5 in AML, as detailed later in **Figure 5**, where it exhibits a negative correlation with similar pathways, underscoring its profound context-dependent functionality.

### 3.4 Immune signature of HOXA5 in pan-cancer

**Figure 6:**
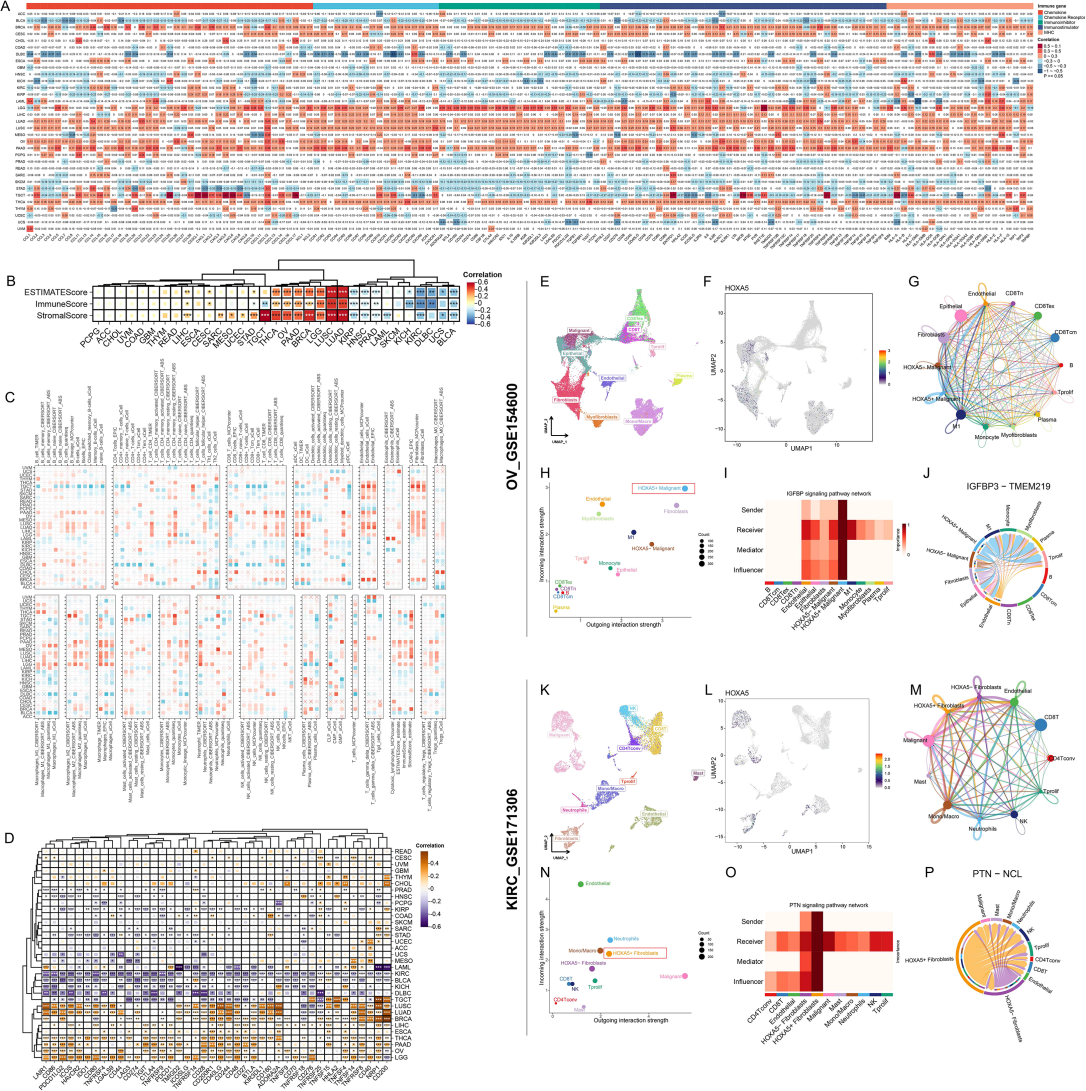
HOXA5 shapes the tumor immune microenvironment through spatially resolved cell-cell communication. **(A)** Correlation between HOXA5 and immune regulators; **(B)** Correlation between HOXA5 and ESTIMATE scores; **(C)** Spearman correlation between HOXA5 and immune cell infiltration (multiple algorithms); **(D)** Correlation between HOXA5 and immune checkpoint expression; **(E, F, K, L)** UMAP plots showing HOXA5 expression in malignant cells and fibroblasts in ovarian cancer (OV, GSE154600) and kidney renal clear cell carcinoma (KIRC, GSE171306); **(G, H, M, N)** Outgoing communication strength of HOXA5+ vs. HOXA5- malignant cells **(G, H)** and fibroblasts **(M, N)**; **(I, J)** Key signaling pathways **(I)** and ligand-receptor pairs **(J)** from HOXA5+ malignant cells; **(O, P)** Key signaling pathways **(O)** and ligand-receptor pairs **(P)** from HOXA5+ fibroblasts. *p < 0.05, **p < 0.01, ***p < 0.001.

HOXA5’s immune associations exhibited tumor heterogeneity. Negative correlations with immune regulators occurred in BLCA, DLBC, SKCM; positive correlations occurred in LUAD, LUSC, THCA (**Figure 6A**). Tumor microenvironment (TME) assessment confirmed this: HOXA5 positively correlated with ImmuneScore/StromalScore in LUAD, LUSC, LGG; negatively correlated in BLCA, KIRC (**Figure 6B**). Multiple algorithms linked HOXA5 to immune infiltrates (e.g., CD8+ T cells, Endothelial cells, Fibroblasts) and immune checkpoints (negative: CD274, PDCD1, CTLA4 in KIRC/BLCA; positive: LUSC/LUAD/BRCA) (**Figures 6C, D**). Single-cell analysis (OV: GSE154600; KIRC: GSE171306) localized HOXA5 primarily to malignant cells and fibroblasts (**Figures 6E, F, K, L**). HOXA5+ malignant cells and HOXA5+ fibroblasts exhibited stronger outgoing communication signals than HOXA5- counterparts (**Figures 6G, H, M, N**). In ovarian cancer HOXA5+ malignant cells, the IGFBP pathway was a key mediator, influencer, sender, and receiver (**Figure 6I**). Ligand-receptor analysis indicated HOXA5+ malignant cells exerted stronger regulation on Fibroblasts, Epithelial, Endothelial, Monocyte, M1, Tprolif, and Plasma cells via IGFBP3-TMEM219 than HOXA5- cells (**Figure 6J**). In KIRC HOXA5+ fibroblasts, the PTN pathway dominated communication roles (**Figure 6O**). HOXA5+ fibroblasts regulated Malignant, Mast, NK, Tprolif, Mono/Macro, Neutrophils, CD4Tconv, CD8T, and Endothelial cells more strongly via PTN-NCL than HOXA5- fibroblasts (**Figure 6P**).

### 3.5 High HOXA5 expression predicts poor prognosis in AML

**Figure 7:**
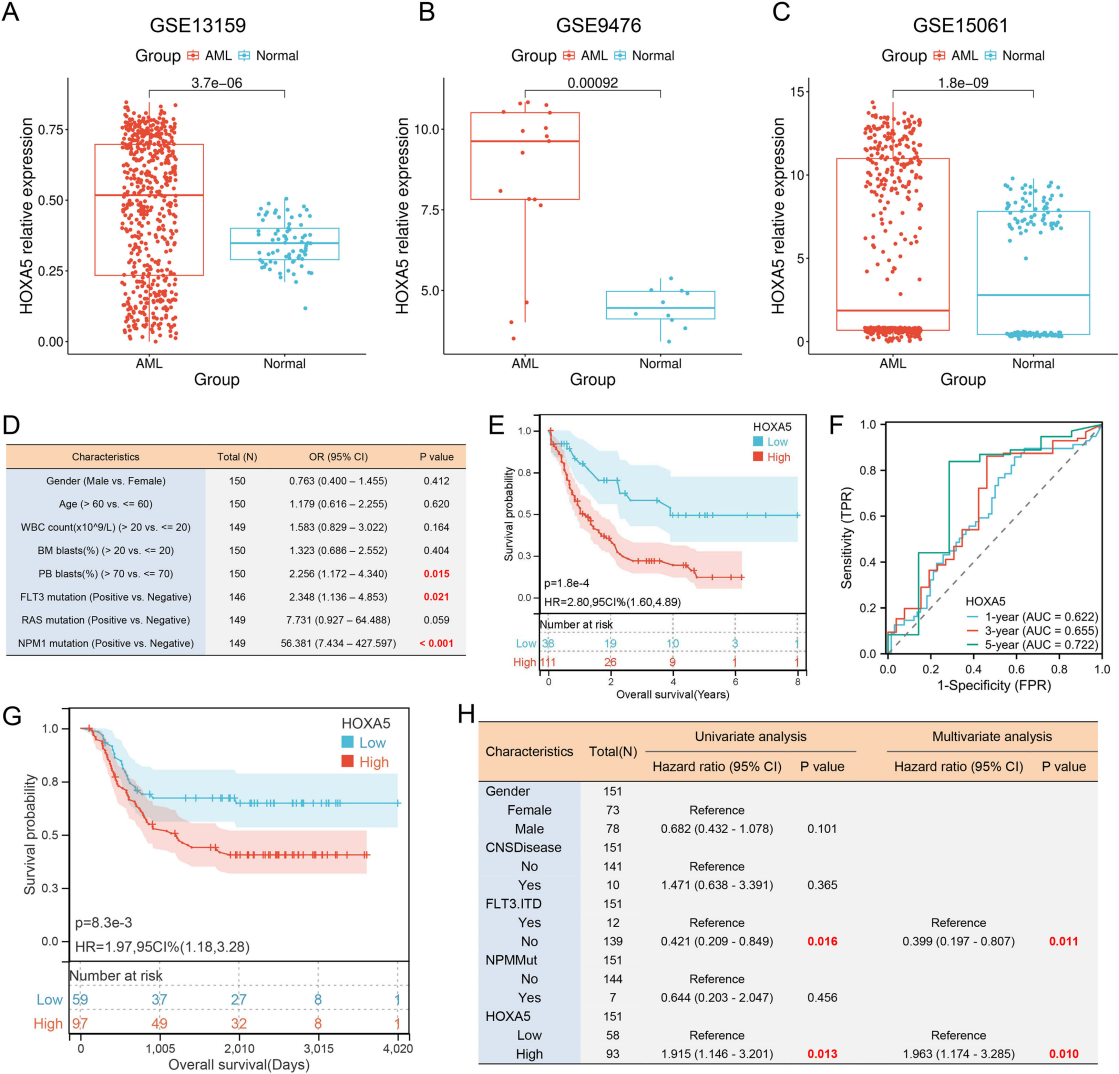
High HOXA5 expression is an independent prognostic factor in AML **(A-C)** Validation of HOXA5 overexpression in AML using GEO datasets; **(D)** Association between HOXA5 expression and AML clinical features (logistic regression); **(E)** Kaplan-Meier overall survival (OS) analysis for HOXA5-high vs. -low AML patients; **(F)** Time-dependent ROC analysis assessing the predictive performance of HOXA5 for 1-, 3-, and 5-year survival in AML patients; **(G)** Validation of prognostic value in the TARGET-AML pediatric cohort; **(H)** Multivariate Cox regression identifying HOXA5 as an independent prognostic factor.

Previous studies have highlighted that HOXA5 is significantly elevated in AML patients (42), and we confirmed this using a public datasets (**Figures 7A-C**). HOXA5 correlated significantly with PB blasts (%), FLT3 mutation, and NPM1 mutation status (**Figure 7D**, **Supplementary Figures S4A-C**). High HOXA5 expression predicted significantly shorter overall survival (OS) in AML patients (**Figure 7E**), a finding further validated using the Kaplan-Meier Plotter database (**Supplementary Figure S4D**). This association was consistently observed across multiple independent cohorts: in the GSE6891 dataset (N = 520), high HOXA5 expression significantly predicted poor OS (HR = 1.81, 95% CI: 1.39–2.35, p = 8.2E-06); in GSE12417 (N = 242), HR = 1.49 (95% CI: 1.07–2.08, p = 0.017); in an additional cohort from GSE12417 (N = 552), HR = 2.01 (95% CI: 1.57–2.57, p = 2.0E-08); in GSE1159 (N = 260), HR = 1.94 (95% CI: 1.37–2.75, p = 1.5E-04); and in GSE8970 (N = 34), HR = 2.36 (95% CI: 1.04–5.36, p = 0.035)(**Supplementary Figures S4E-I**). Restricted cubic spline (RCS) analysis indicated a linear relationship between HOXA5 expression and AML death risk (**Supplementary Figure S4J**). Patients in the highest HOXA5 expression quartile (Q1) had the highest mortality (**Supplementary Figure S4K**). Time-dependent ROC analysis showed AUC values of 0.622 (1-year), 0.655 (3-year), and 0.722 (5-year) (**Figure 7F**). Analysis based on the TARGET-AML (n = 156) dataset showed that high HOXA5 also predicted poor OS in childhood AML (HR = 1.97, 95% CI: 1.18–3.28, p = 8.3E-03) (**Figure 7G**). Multivariate Cox regression confirmed HOXA5 as an independent prognostic factor (**Figure 7H**). A nomogram incorporating HOXA5 and clinical features predicted 1-, 3-, and 5-year survival (**Supplementary Figure S4L**). Calibration and decision curve analysis (DCA) confirmed model accuracy for 3- and 5-year predictions (**Supplementary Figures S4M-O**).

### 3.6 Immunological characteristics of HOXA5 in AML

**Figure 8:**
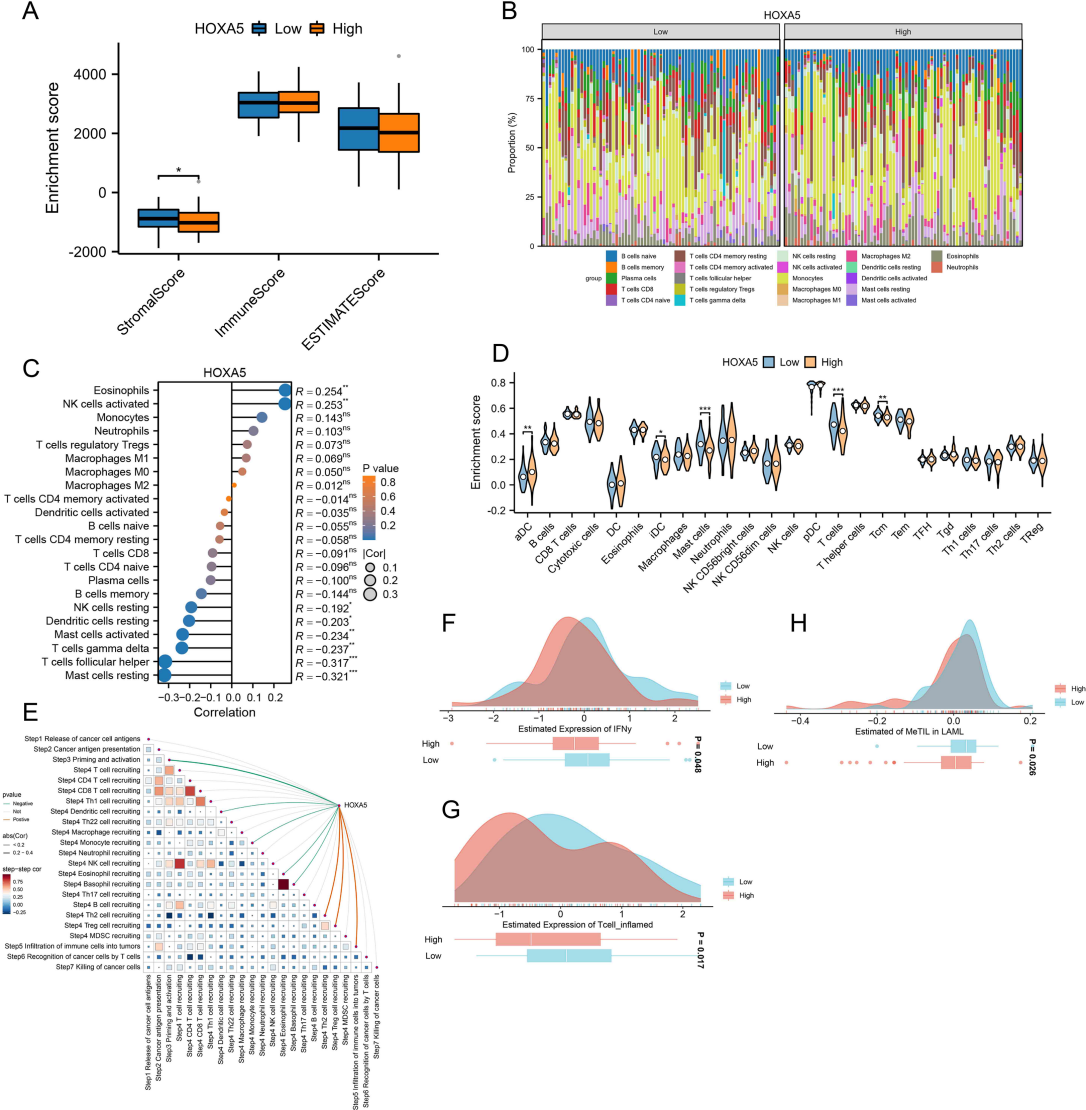
Immunological features associated with HOXA5 expression in AML. **(A)** ESTIMATE scores in HOXA5-high vs. -low groups; **(B)** Differential immune cell infiltration (CIBERSORT); **(C)** Correlation between HOXA5 expression and immune cell infiltration levels; **(D)** ssGSEA analysis of immune cell infiltration; **(E)** Correlation between HOXA5 and TIP (Tumor Immunophenotype) scores; **(F-H)** Comparison of IFNγ score **(F)**, T-cell inflamed score **(G)**, and MeTIL score **(H)** between HOXA5 expression groups. *p < 0.05, **p < 0.01, ***p < 0.001.

ESTIMATE analysis revealed significantly lower StromalScores in the HOXA5-low group (**Figure 8A**). Immune infiltration differed between groups: HOXA5-high showed increased eosinophils and activated NK cells; HOXA5-low showed increased resting mast cells, Tfh cells, and γδ T cells (**Figure 8B**), validated by correlation analysis (**Figure 8C**). ssGSEA indicated enhanced infiltration of iDCs, mast cells, pDCs, and Tcm cells in the HOXA5-low group (**Figure 8D**). TIP database analysis showed HOXA5 expression positively correlated with Th2 recruitment, Treg recruitment, and general immune infiltration, but negatively correlated with priming and activation (**Figure 8E**). Notably, the HOXA5-low group exhibited higher IFNγ scores, T cell-inflamed scores, and MeTIL scores (**Figures 8F-H**).

### 3.7 HOXA5 regulates AML cell proliferation and cell cycle

**Figure 9:**
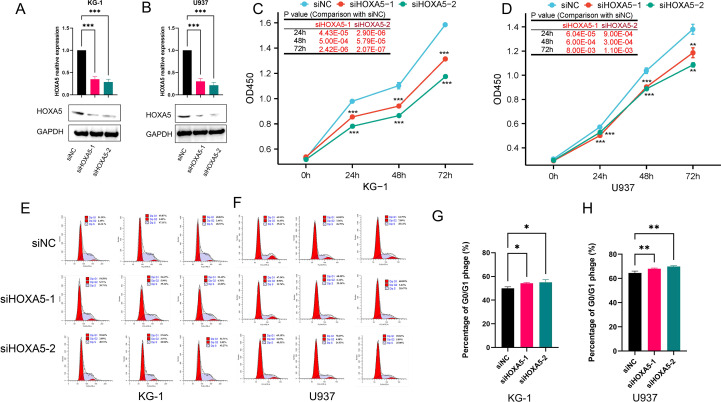
HOXA5 knockdown inhibits proliferation and induces cell cycle arrest in AML cells. **(A, B)** Knockdown efficiency of two independent siRNAs targeting HOXA5 in KG-1 and U937 cells, validated by RT-qPCR **(A)** and Western blot **(B)**; **(C, D)** Cell proliferation (CCK-8 assay) after HOXA5 knockdown; **(E-H)** Cell cycle distribution analyzed by flow cytometry after HOXA5 knockdown. Representative plots **(E, F)** and quantification of G0/G1 phase **(G, H)** are shown. *p < 0.05, **p < 0.01, ***p < 0.001.

Functional studies in AML cell lines (U937, KG-1) utilized two independent siRNAs. Efficient HOXA5 knockdown was confirmed by RT-qPCR and Western blot (**Figures 9A, B**). HOXA5 loss significantly inhibited proliferation (CCK-8 assay; **Figures 9C, D**) and induced G0/G1 cell cycle arrest (**Figures 9E-H**), demonstrating its critical role in AML cell cycle regulation and proliferation.

### 3.8 RNA-seq analysis of HOXA5 mechanisms in AML

Genomic analysis revealed HOXA5 interactions with TWIST1, HOXA4, and NCAM1 (**Supplementary Figure S5A**). Protein interaction networks connected HOXA5 to HOXB4, HOXC6, and HOXC4 (**Supplementary Figure S5B**). Comparing HOXA5-high vs. HOXA5-low groups identified 1,494 dysregulated genes (610 up, 884 down; **Figure 5A**). WGCNA identified the MEdarkolivegreen module as most correlated with HOXA5 (**Figure 5B**, **Supplementary Figure S5C**). Cross-analysis yielded 45 core positively correlated genes (**Figure 5C**). GO-BP enrichment implicated HOXA5 in anterior/posterior patterning and embryonic skeletal development. GO-MF enrichment highlighted DNA-binding transcription activator activity (RNA Pol II-specific) and growth factor activity (**Figure 5D**). GSVA showed negative correlations between HOXA5 and angiogenesis/metastasis pathways (**Figure 5E**). This inverse relationship in AML diverges from the positive associations between HOXA5 and these pathways observed in the pan-cancer analysis of solid tumors (**Figure 4**), highlighting a fundamental difference in HOXA5’s mechanistic role between solid and hematological malignancies. Metabolic analysis indicated activation of valine, leucine, and isoleucine biosynthesis in HOXA5-high AML (**Figure 5F**, **Supplementary Figure S5D**). GSEA revealed enrichment of focal adhesion, ECM-receptor interaction, and primary immunodeficiency pathways in HOXA5-low AML (**Figure 5G**).

RNA-seq in HOXA5-knockdown KG-1 cells identified 566 DEGs (siHOXA5-1: 300 up, 266 down; **Figure 5H**) and 591 DEGs (siHOXA5-2: 317 up, 274 down; **Figure 5I**) (**Supplementary Tables S3, 4**). Intersection yielded 442 core HOXA5-regulated DEGs (**Figure 5J**). Enrichment analysis linked HOXA5 to cholesterol biosynthesis, cell-cell adhesion, ECM, immune suppression, and ECM-receptor interaction (**Figures 5K, L**), consistent with bioinformatics findings.

### 3.9 Association between HOXA5 expression and drug sensitivity

**Figure 10:**
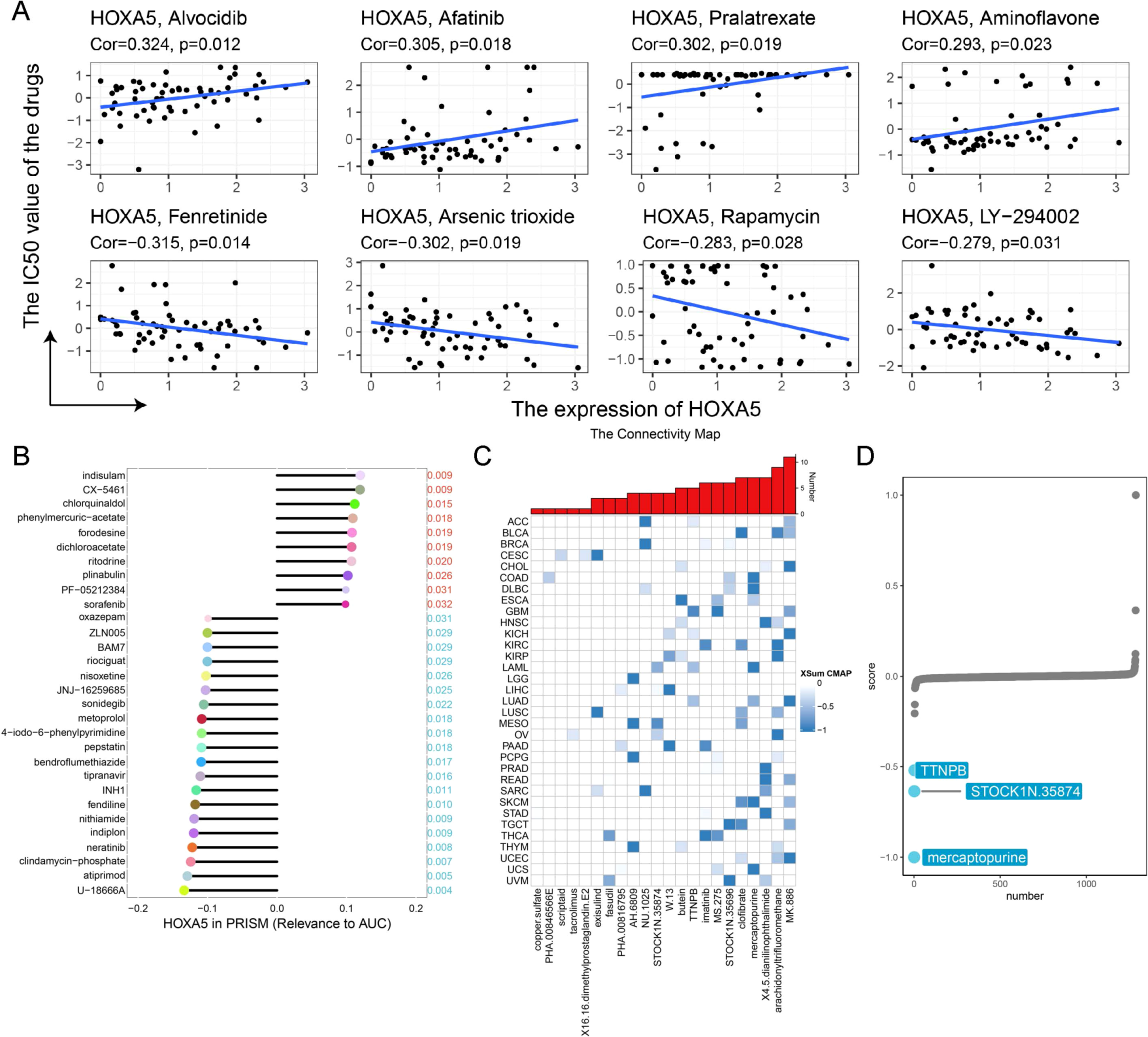
Pharmacogenomic analysis identifies mercaptopurine as a potential therapeutic agent targeting HOXA5. **(A)** Correlation between HOXA5 expression and drug sensitivity (IC50) in the CellMiner database; **(B)** Correlation between HOXA5 expression and drug sensitivity (AUC) in the PRISM repurposing dataset; **(C)** Candidate compounds from Connectivity Map (CMap) predicted to reverse HOXA5 high-expression signatures; **(D)** Negative correlation between HOXA5 expression and mercaptopurine sensitivity in AML.

CellMiner analysis revealed positive correlations between HOXA5 and sensitivity to Alvocidib (CDK inhibitor), Afatinib (EGFR inhibitor), Pralatrexate (antifolate), and Aminoflavone (Aryl hydrocarbon Receptor agonist). Negative correlations occurred with Fenretinide (retinoid), Arsenic trioxide, Rapamycin (mTOR inhibitor), and LY-294002 (PI3K inhibitor) (**Figure 10A**). PRISM analysis confirmed negative correlations with U-18666A (cholesterol synthesis inhibitor), atiprimod (JAK2 inhibitor), and neratinib (HER2 inhibitor) (**Figure 10B**). cMAP analysis identified MK-886 (FLAP inhibitor) as a potential countermeasure against HOXA5-mediated oncogenesis (**Figure 10C**). Mercaptopurine (purine antagonist) showed the strongest negative correlation in AML (**Figure 10D**) among the compounds analyzed, suggesting it might possess potential efficacy against HOXA5-driven leukemia. To explore a potential mechanism, we performed molecular docking studies to predict the HOXA5-mercaptopurine interaction. SWISS-MODEL alignment identified PDB 2R5Z as the optimal template (**Supplementary Figure S6A**) for HOXA5 tertiary structure modeling (**Supplementary Figure S6B**). Model validation via Ramachandran plot showed 92.1% of residues in favored regions (**Supplementary Figure S6C**). HOXA5 residues Thr200, Tyr202, and Gln238 were predicted to form a hydrogen bond network that could potentially stabilize the drug complex (**Supplementary Figures S6D-F**). This computational model suggests a possible direct interaction, which remains to be validated experimentally.

## 4 Discussion

While HOXA5 acts as a tumor suppressor in breast cancer, lung adenocarcinoma, and cervical cancer (8, 43, 44), it exhibits oncogenic properties in glioma (11). This pan-cancer study systematically characterized HOXA5’s expression patterns, immune interactions, clinical significance, and mechanistic role in AML using multi-omics and functional validation. We reveal, for the first time, its spatially resolved regulatory network within the tumor immune microenvironment and functional heterogeneity between solid and hematologic malignancies, offering novel perspectives for targeted intervention (45).

As a developmental transcription factor, HOXA5 typically functions as a tumor suppressor in solid tumors. We confirmed significant downregulation in BRCA, LUAD, and COAD, where low expression correlated with poor prognosis, consistent with literature (46, 47). Crucially, we uncovered HOXA5’s oncogenic potential at a pan-cancer scale—its elevated expression in GBM and AML promotes malignancy. This functional duality highlights the context-dependency of HOXA5’s biological effects. Single-cell analysis revealed that HOXA5+ malignant cells enhance communication with fibroblasts and endothelial cells via IGFBP3-TMEM219 signaling, while HOXA5+ fibroblasts reshape the immune microenvironment via PTN-NCL, providing mechanistic insights into its tumor-promoting roles.

DNA methylation critically regulates HOXA5. We confirmed promoter hypermethylation in cancers like BRCA and LUAD (48–50), but also revealed tumor-type specificity in methylation-expression relationships: negative in KICH and SARC versus positive in CHOL and TGCT. This bidirectional regulation suggests involvement of enhancer methylation or demethylase activity beyond promoter silencing (50). eQTL-GWAS colocalization linked rs3757640 to cancer risk, supporting genetic-epigenetic interplay in HOXA5 regulation.

HOXA5 displays contradictory immunoregulatory roles: positively correlating with immune infiltration in LUAD/LUSC but negatively in BLCA/KIRC. This divergence likely stems from tissue-specific downstream pathways. GSVA linked high HOXA5 to angiogenesis and EMT. HOXA5+ cells recruit immunosuppressive cells via factors like IGFBP3. Single-cell communication analysis showed HOXA5+ malignant cells enhance IGFBP signaling and Treg recruitment, aligning with its inhibition of T cell infiltration in AML. Elevated IFNγ and T cell inflammation scores in HOXA5-low AML suggest it may predict immunotherapy resistance, potentially explaining differential PD-1 inhibitor efficacy in HOXA5-high tumors (51).

HOXA5 is highly expressed in AML and promotes proliferation, consistent with its pro-tumorigenic role (52). High HOXA5 independently predicts poor AML prognosis and cooperates with FLT3/NPM1 mutations. Functional analysis revealed that HOXA5 maintains leukemia through metabolic reprogramming (cholesterol biosynthesis) and ECM remodeling. HOXA5 knockdown dysregulated ECM-receptor interactions and downregulated adhesion molecules, suggesting it sustains malignancy within the protective bone marrow niche via integrin-mediated survival signals. Notably, we observed that HOXA5 exhibited positive correlations with angiogenesis, EMT, and stemness in our pan-cancer analysis—likely driven by its role in solid tumors—yet showed a negative association with angiogenesis and metastasis pathways in AML. This apparent dichotomy underscores the profound context-dependency of HOXA5’s function. In solid tumors, HOXA5 may facilitate tumor progression by enhancing vascularization, cellular plasticity, and stemness. In contrast, within the AML microenvironment, HOXA5 appears to sustain leukemogenesis through distinct mechanisms, principally metabolic reprogramming involving cholesterol biosynthesis and alterations in ECM-receptor interactions, as evidenced by our functional genomics and knockdown experiments. This divergence emphasizes the tissue-specific regulatory networks orchestrated by HOXA5 and solidifies its characterization as a dual-function regulator in cancer biology. Enrichment of immune escape signals (e.g., impaired negative regulation of immunity) corroborated HOXA5-driven Treg recruitment and IFNγ suppression, indicating dual roles in cell-intrinsic proliferation (via G0/G1 escape) and cell-extrinsic immunosuppression. Notably, elevated IFNγ and T cell inflammation scores in HOXA5-low AML suggest that HOXA5 may serve as a potential predictor of immunotherapy resistance. However, it is important to note that these findings are based on bioinformatic associations and have not been validated in an independent cohort of ICI-treated patients. Therefore, we refrain from making causal claims regarding HOXA5 and ICI efficacy. Future studies should directly investigate the relationship between HOXA5 expression and response to immune checkpoint inhibitors in prospective or publicly available ICI-treated cohorts to validate its potential as a predictive biomarker.

Limitations: TCGA data carry sample selection bias; prospective validation is needed. Furthermore, while we integrated TCGA and GTEx data using Z-score normalization to mitigate systematic differences, we did not apply explicit batch correction algorithms (e.g., ComBat) or control for clinical covariates during this integration. Although the consistent dysregulation patterns of HOXA5 observed across multiple independent validation datasets (e.g., GENT2, GEO cohorts) strengthen our findings, we cannot fully rule out that residual batch effects may have influenced the magnitude of the observed expression differences in the combined TCGA-GTEx analysis. The specific effectors of HOXA5-regulated IGFBP3-TMEM219/PTN-NCL axes require experimental validation. Additionally, the binary classification of spatial microregions based on tumor cell content (>0% vs. 0%) may introduce purity-related biases. Cell lines incompletely model patient tumor heterogeneity; primary AML validation is essential. Furthermore, the drug sensitivity analysis identified mercaptopurine as a candidate agent correlated with HOXA5 expression. While molecular docking provided a hypothetical model for a direct interaction, this study lacks experimental validation (e.g., by surface plasmon resonance or cellular thermal shift assays) to confirm that HOXA5 is a direct molecular target of mercaptopurine. The observed correlation could also be mediated through indirect mechanisms or off-target effects.

Future Directions: Analyze HOXA5’s 3D genomic regulatory network using chromatin conformation capture. Explore relationships between HOXA5 expression and immune checkpoint inhibitor (ICI) efficacy. Design allosteric inhibitors based on HOXA5-thiopurine complex structures. Conduct AML intervention trials targeting HOXA5 (e.g., RNA nanomedicines, epigenetic modulators). Of note, although we developed a prognostic nomogram incorporating HOXA5 expression and clinical variables, its generalizability requires further validation in an independent cohort with complete clinical annotation. Future prospective studies are necessary to clarify the clinical utility of this model.

HOXA5 has a “dual personality” in pan-cancer: it is mostly a tumor suppressor in solid tumors, but it is transformed into a tumor promoter in blood tumors. Through multi-omics integration and functional verification, this study revealed for the first time its pivotal role in the tumor space immune microenvironment and its therapeutic target value in AML, laying the foundation for the development of precision treatment based on HOXA5 molecular typing.

## 5 Conclusion

HOXA5 exhibits a “dual personality” in pan-cancer: primarily tumor-suppressive in solid tumors but oncogenic in hematologic malignancies. Through multi-omics integration and functional validation, this study unveils its pivotal role in the spatial tumor immune microenvironment and establishes its therapeutic target value in AML, laying the groundwork for HOXA5-based precision therapies.

## Data Availability

The datasets presented in this study can be found in online repositories. The names of the repository/repositories and accession number(s) can be found in the article/**Supplementary Material**.
