# Supplementary material for: HOXA5-mediated spatial remodeling of tumor-immune interfaces across cancers promotes AML pathogenesis
Source: Front Immunol. 2025 Oct 23;16:1677713. doi: 10.3389/fimmu.2025.1677713 (PMC12589088; doi:10.3389/fimmu.2025.1677713)
Supplement: Supplementary file 1 [file Presentation1.pptx]

## Slide 1
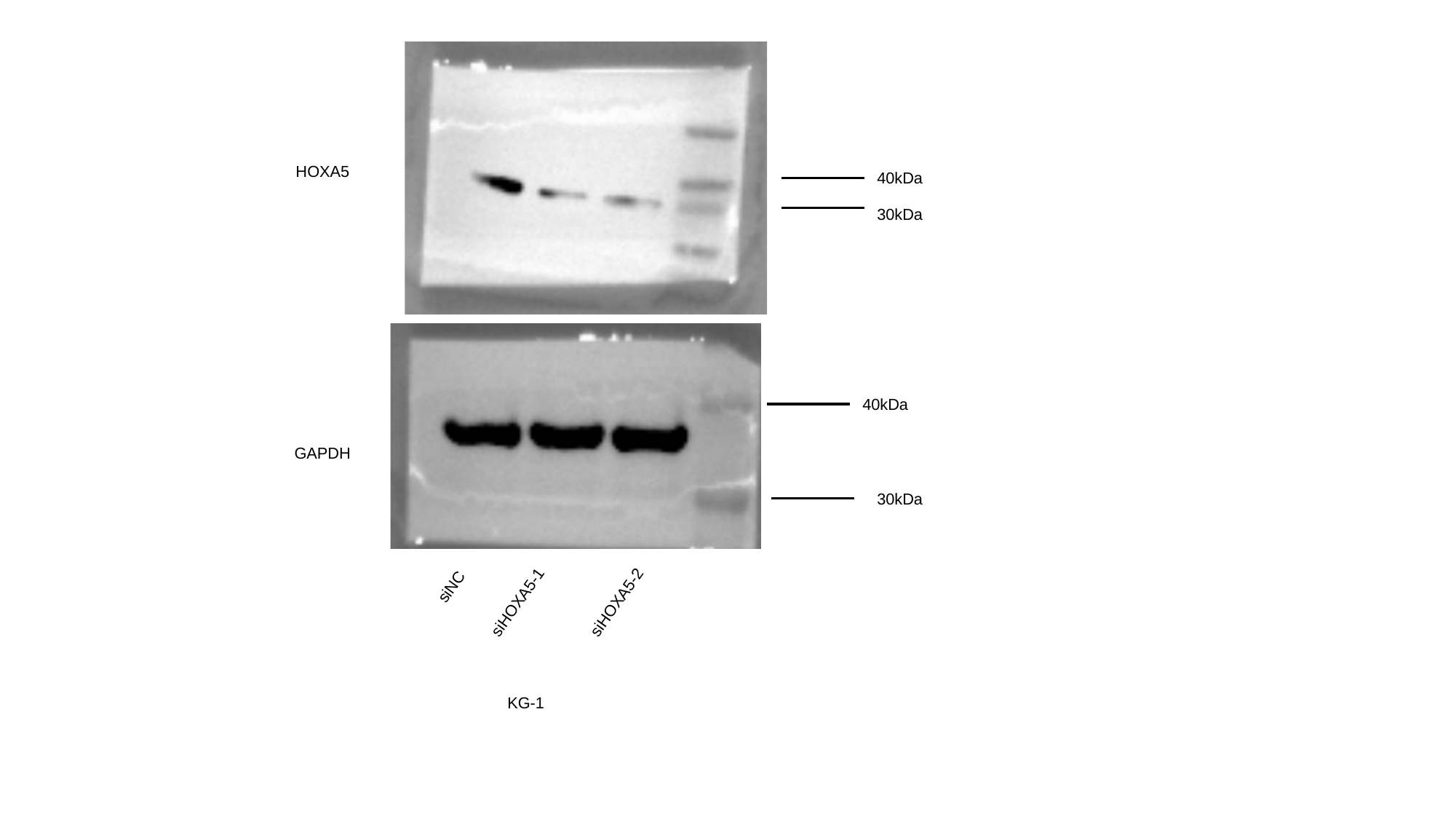

HOXA5
40kDa
30kDa
40kDa
GAPDH
30kDa
siNC
siHOXA5-2
siHOXA5-1
KG-1

## Slide 2
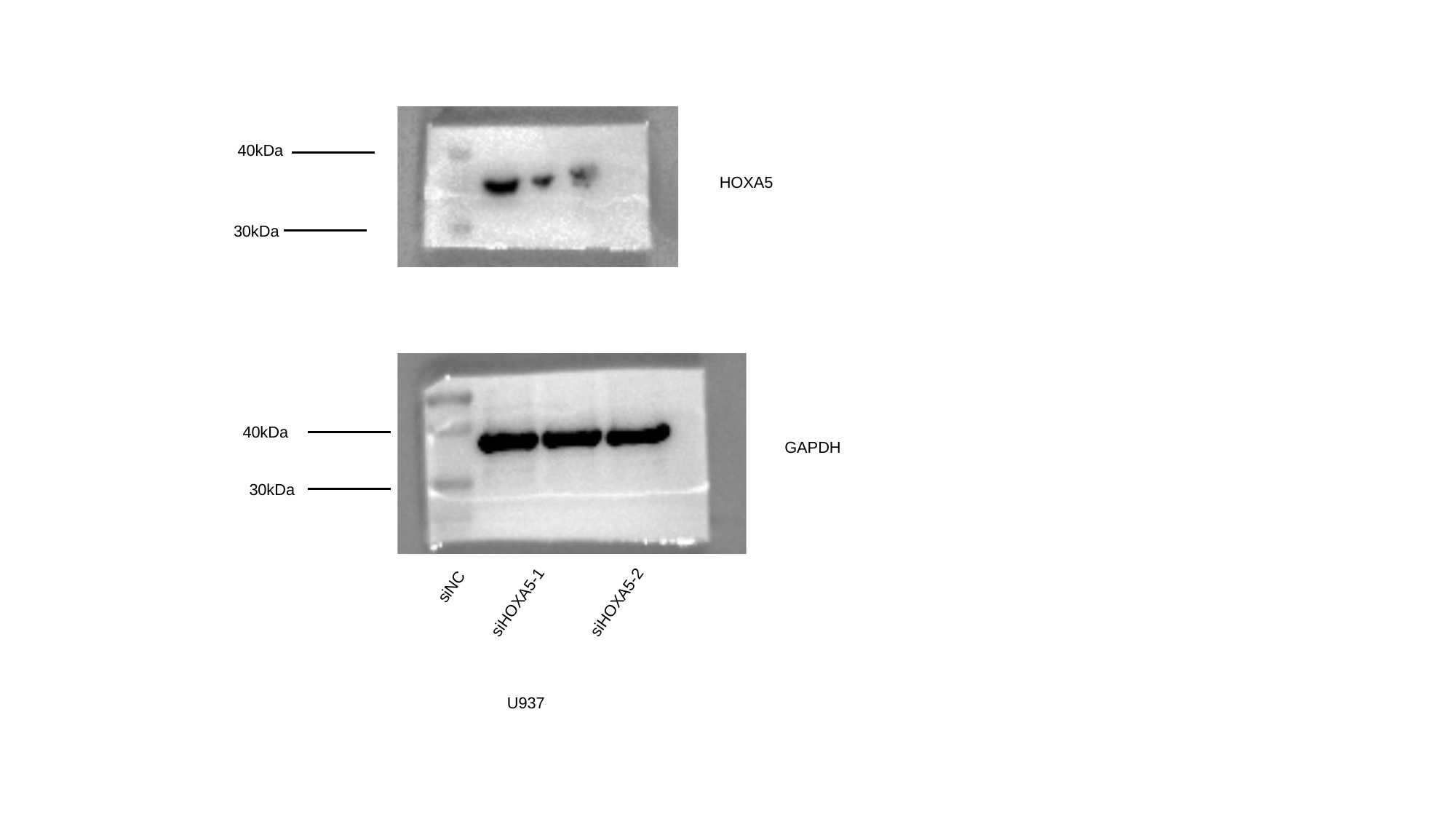

40kDa
HOXA5
30kDa
40kDa
GAPDH
30kDa
siNC
siHOXA5-2
siHOXA5-1
U937
