# Supplementary material for: HOXA5-mediated spatial remodeling of tumor-immune interfaces across cancers promotes AML pathogenesis
Source: Front Immunol. 2025 Oct 23;16:1677713. doi: 10.3389/fimmu.2025.1677713 (PMC12589088; doi:10.3389/fimmu.2025.1677713)
Supplement: Supplementary file 3 [file Table2.docx]

Supplementary Material

HOXA5-Mediated Spatial Remodeling of Tumor-Immune Interfaces Across Cancers Promotes AML Pathogenesis

# Supplementary Figures



**Supplementary Figure 1**. (A) Expression landscape of HOXA5 in human normal tissues. (B) HOXA5 expression patterns in immune cell populations. (C) Expression levels of HOXA5 across tumor cell lines. (D) Analysis of differential expression of HOXA5 transcripts between tumor samples and normal samples. (E) Validation of differential expression of HOXA5 between tumor and normal tissues using the GENT2 database. **p* < 0.05, ***p* < 0.01, ****p* < 0.001, *****p* < 0.0001.

**
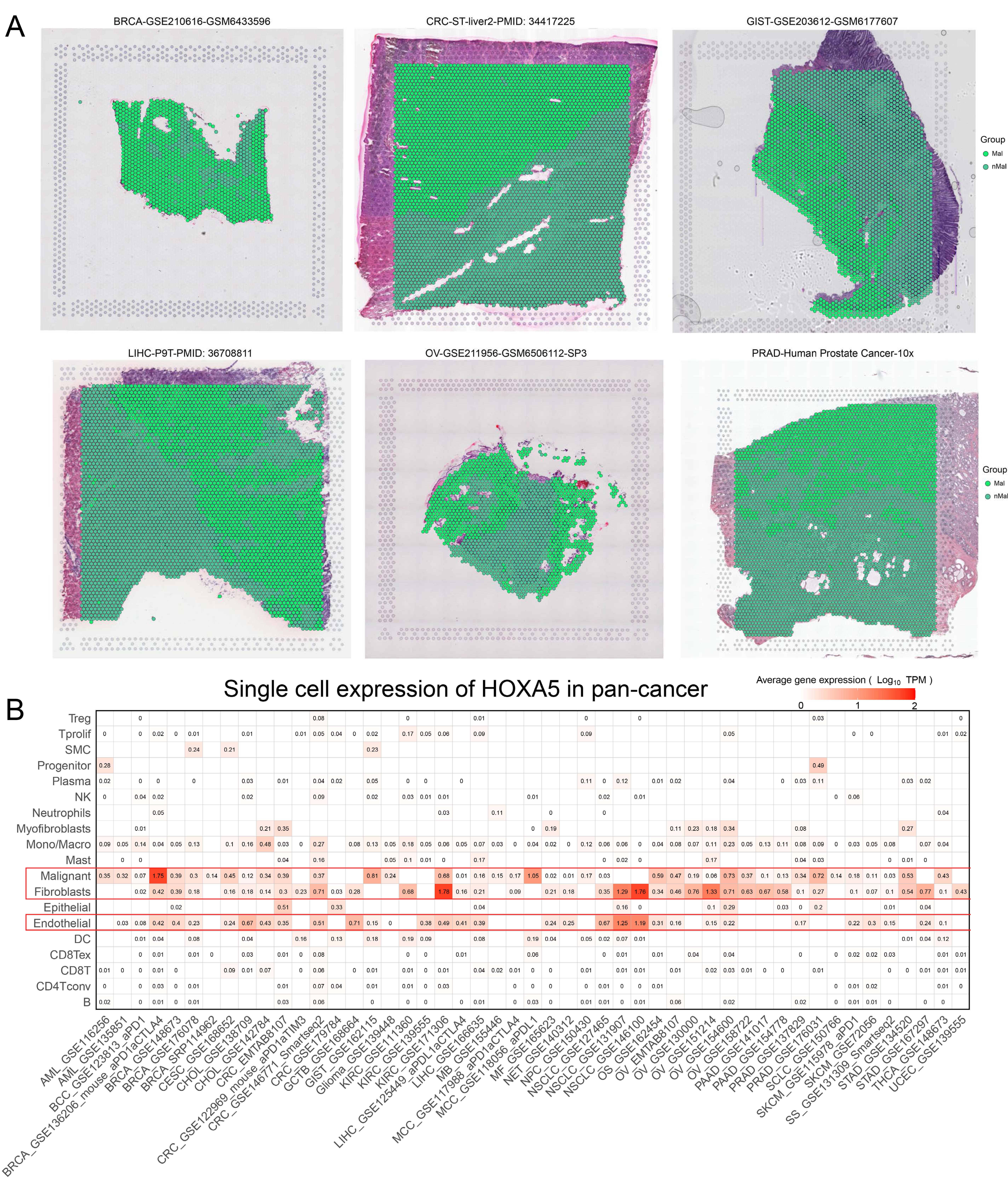
**

**Supplementary Figure 2**. (A) Classification of tissue regions into malignant (Mal) and non-malignant (nMal) based on the proportion of malignant cells in each microregion; (B) Single-cell expression analysis of HOXA5 in pan-cancer.

**
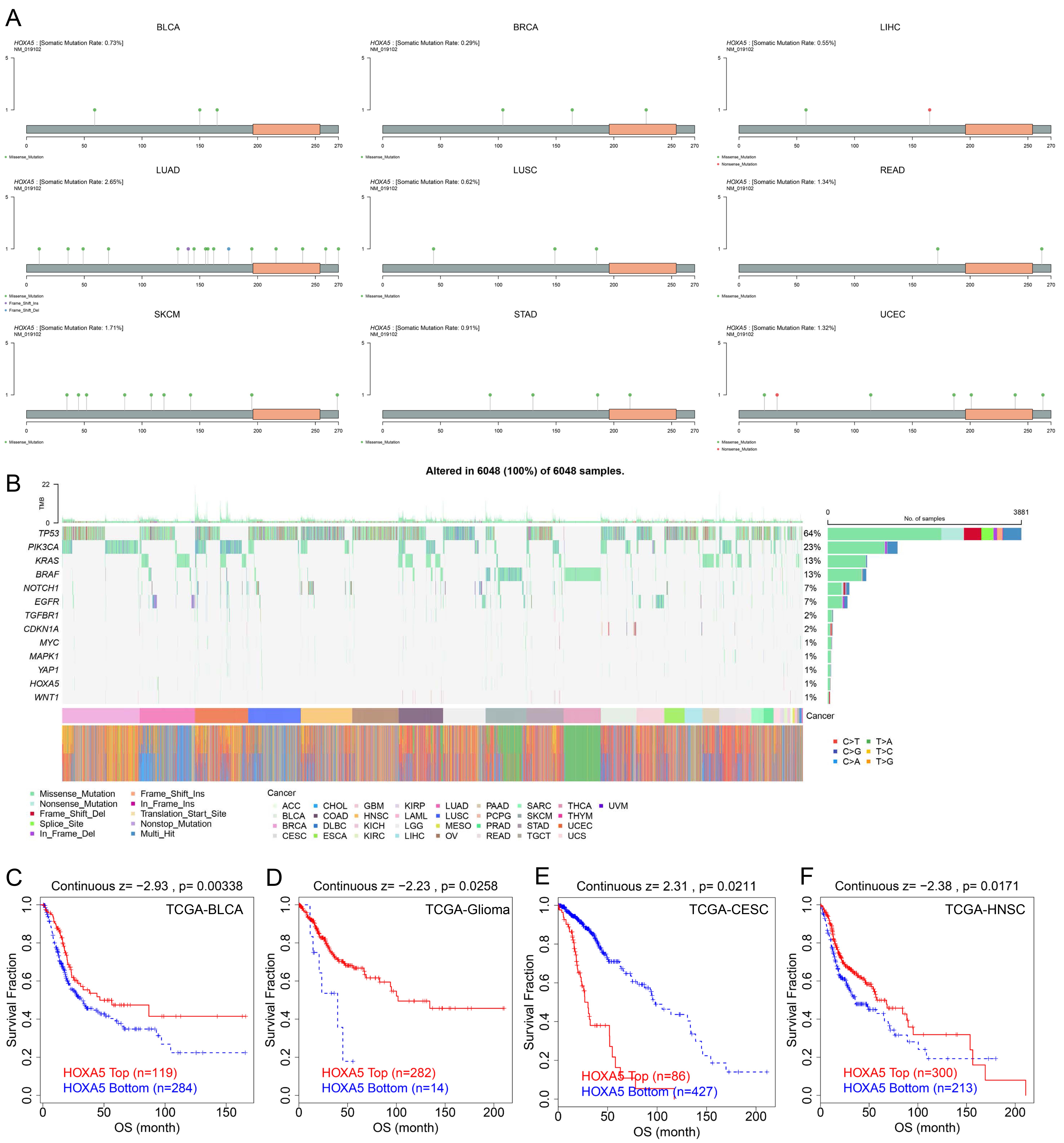
**

**Supplementary Figure 3**. (A) Map of mutation sites of HOXA5 gene in multiple species; (B) HOXA5 mutation waterfall diagram in pan-cancer; (C-F) Association between HOXA5 methylation and tumor prognosis.

**
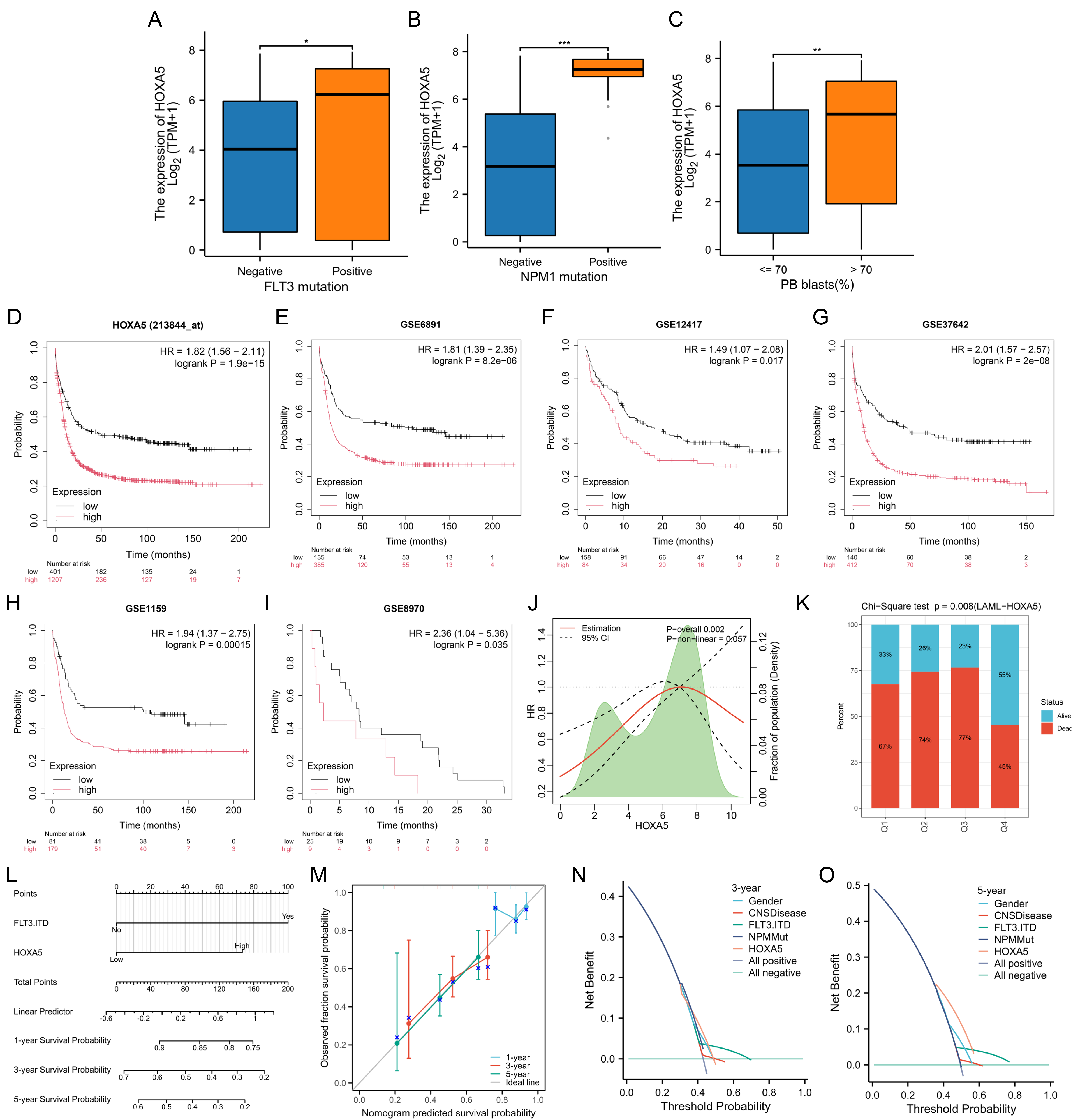
**

**Supplementary Figure 4**. (A-C) Association between HOXA5 expression and AML clinical features: FLT3 mutation status, NPM1 mutation status, and PB blasts percentage; (D-I) Kaplan-Meier survival analysis (via Kaplan-Meier Plotter) demonstrating the association between high HOXA5 expression and poor prognosis in AML; (J) Restricted Cubic Splines (RCS) analysis evaluating the non-linear relationship between HOXA5 expression levels and overall survival (OS) in AML; (K) OS analysis stratified by HOXA5 expression quartiles (Q1 highest, Q4 lowest); (L) Nomogram integrating HOXA5 and clinical features for predicting 1-, 3-, and 5-year survival; (M) Calibration curve of the nomogram; (N-O) Decision curve analysis (DCA) for the 3- and 5-year survival predictions. **p* < 0.05, ***p* < 0.01, ****p* < 0.001.

**
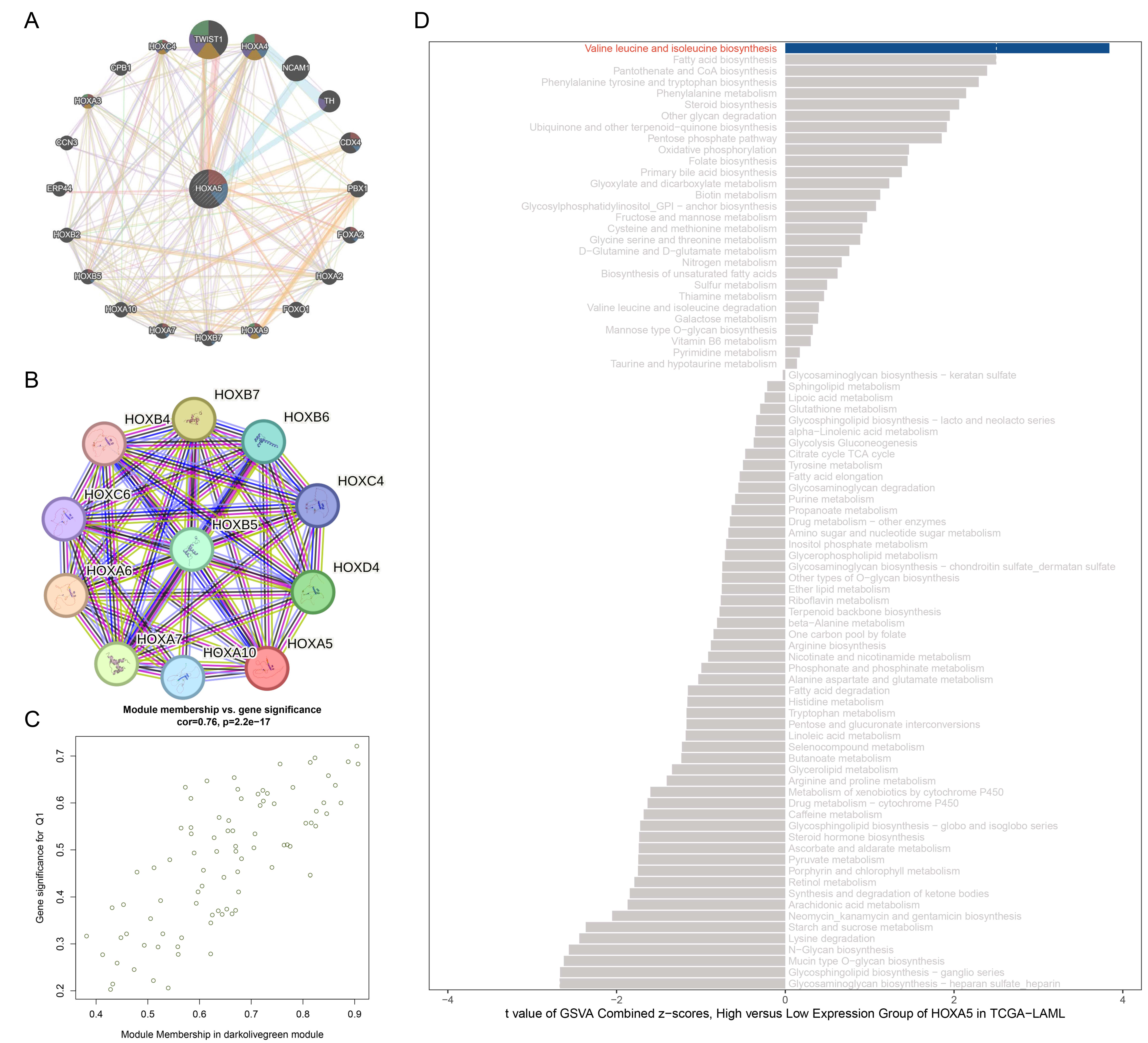
**

**Supplementary Figure 5**. (A) Gene interaction network of HOXA5; (B) Protein-protein interaction (PPI) network of HOXA5; (C) Correlation between Q1 group (highest HOXA5 expression) and the darkolivegreen module; (D) Differential GSVA scores of metabolic pathways between HOXA5 high- and low-expression groups.


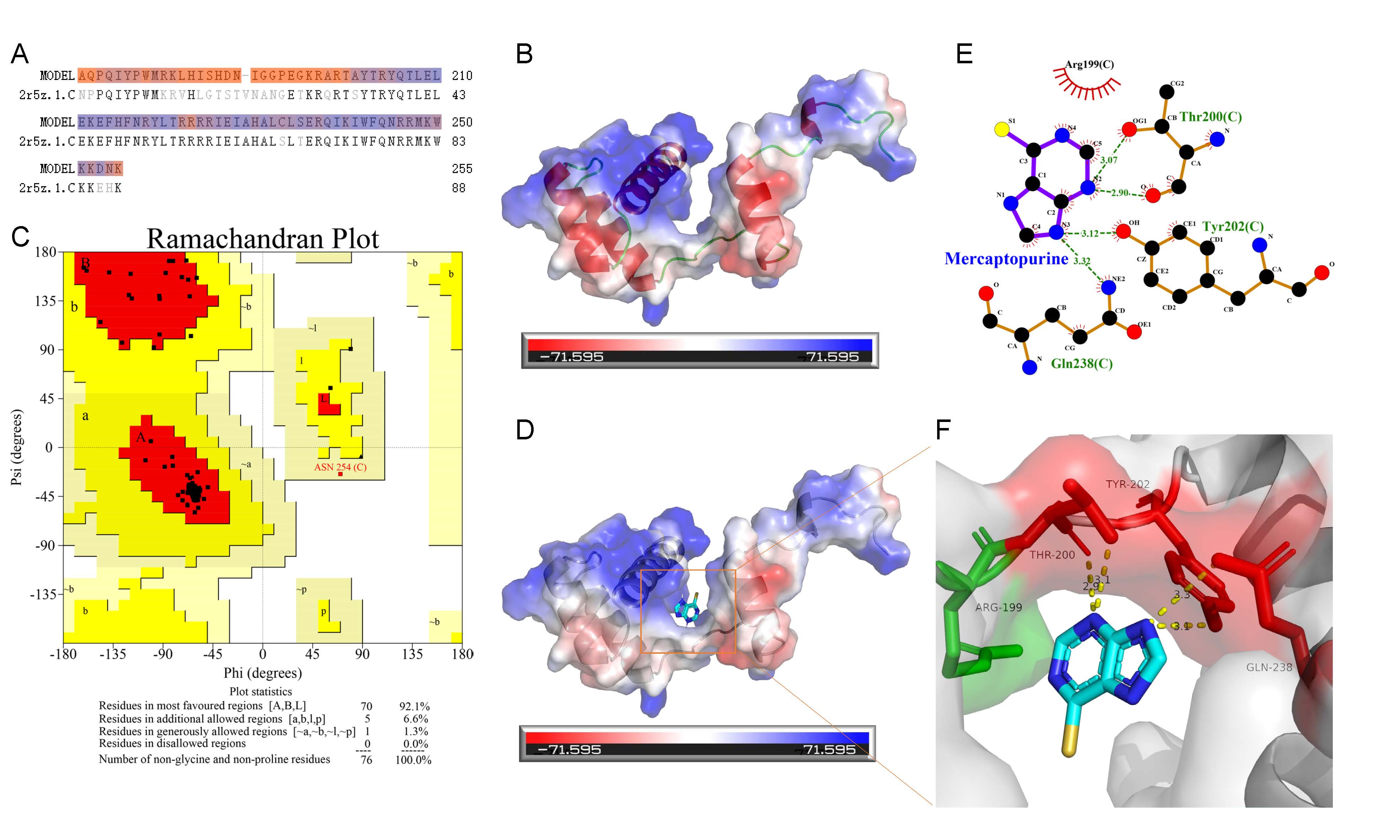


**Supplementary Figure 6**. (A) Amino acid sequence alignment between HOXA5 and its homology template (PDB: 2R5Z); (B) Predicted tertiary structure of HOXA5 from SWISS-MODEL; (C) Ramachandran plot assessing the quality of the HOXA5 structural model; (D) Predicted binding pose of mercaptopurine within the HOXA5 binding pocket (CB-Dock2); (E) 2D ligand-protein interaction diagram; (F) 3D structural representation of the HOXA5-mercaptopurine complex.
